# Supplementary material for: Diketopyrrolopyrrole-based two-dimensional poly(arylene vinylene)s with high charge carrier mobility
Source: Nat Commun. 2026 Feb 3;17:1348. doi: 10.1038/s41467-026-69061-4 (PMC12873148; doi:10.1038/s41467-026-69061-4)
Supplement: Supplementary file 1 — Supplementary Information [file 41467_2026_69061_MOESM1_ESM.pdf]

Supplementary Information

Table of Contents

Section A. Methods

A1. Instrumentation and Experimental Details

A2. DFT Calculation Details

Section B. Materials and Synthetic Procedures

Section C. Supporting Figures

Section D. Supporting Tables

Section E. References

Section A. Methods

A1. Instrumentation and Experimental Details

Nuclear magnetic resonance (NMR) spectroscopy. Solution-state ^{1}H NMR spectra were measured on a BRUKER AVANCE III 300 MHz spectrometer using a single pulse experiment with 13.3 µs and 10 µs at 30-degree pulse length. Solid-state MAS NMR spectra were acquired by a Bruker Advance 800 MHz spectrometer and a commercially available 1.3 mm double-resonance magic angle spinning (MAS) probe at 50 kHz. ^{1}H Depth MAS spectra were recorded at 2 µs pulse length and 3 s delay time. The probe background signal is suppressed by using a Depth pulse sequence. For the ^{13}C spectrum, 60 K scans are collected at a delay time of 3 s (more than five times of ^{1}H-T_1) and 3 ms and 50 µs contact time with a ramped ^{1}H decoupled (SPINAL-64) cross-polarization (CP) pulse sequence at a resonance frequency of 201.23 MHz. Short contact time ^{13}C-NMR spectra suppressed signal from quaternary and mobile aliphatic ^{13}C resonance, thereby assisting in the assignment of the spectrum. ^{13}C spectra are simulated by using ACD/Labs software. All spectra were referenced to tetramethylsilane (TMS), used as a secondary reference for ^{13}C, resonating at 29.5 ppm. ^{1}H–^{13}C HETCOR spectra were acquired at 800 MHz. ^{1}H resonance frequency and a 50 kHz sample spinning rate using ^{13}C detection, i.e., the well-resolved ^{13}C dimension was directly detected with 128 T_1 increments. Heteronuclear polarization transfer was achieved using ramped cross-polarization with 1 ms CP contact time. Overall, 200 scans were accumulated.

Powder X-ray diffraction (pXRD) analysis. pXRD patterns were obtained on a STOE STADI P diffractometer with Cu Kα line focused radiation at 40 kV and 40 mA from 2θ in the range of 2° to 40° with 0.02° increment.

Fourier-transform infrared (FT-IR) spectroscopy. FT-IR spectra were collected on a Bruker Tensor II spectrometer with a universal Zn-Se ATR (attenuated total reflection) accessory ranging from 400 cm^{-1} to 4000 cm^{-1}.

Thermogravimetric analysis (TGA). TGA from 20 to 1000 °C was carried out on a TG50 analyzer (Mettler-Toledo) in a nitrogen atmosphere using a 10 °C/min ramp without equilibration delay.

Scanning electron microscopy (SEM). SEM (FESEM, Zeiss Gemini 500) was used to investigate the morphology of the as-synthesized 2D PAVs at an accelerating voltage of 3.0 and 3.5 kV. Powder samples were dispersed in ethanol and then dropped onto silicon substrates, which were attached to a flat aluminum sample holder using conductive adhesive tapes and then coated with gold.

High-resolution transmission electron microscopy (HR-TEM). HR-TEM images were collected using a JEOL JEM F200 operating at 200 kV.

Nitrogen physisorption measurements. N_2 physisorption isotherms were recorded by a Micrometrics ASAP 2020-M surface area analyzer up to 1 bar at 77 K. Before measurements, samples were activated for 12 h at 120 °C. The pore size distribution was analyzed using the nonlocal density functional theory (NLDFT) method.

Ultraviolet photoelectron spectroscopy (UPS). The samples were transferred to an ultrahigh vacuum chamber (ESCALAB Xi+ by Thermo Scientific, base pressure: $2 * 10^{-10}$ mbar) for UPS measurements. UPS measurements were carried out using a He discharge lamp ($h\nu$ = 21.2 eV) and a pass energy of 2 eV.

Chemical stability test. The samples were dispersed in different solvents, such as THF, DMF, NMP, HCl (37%, 12 M), and NaOH (12 M), for 24 hours. Before FT-IR spectra and pXRD measurement, precipitates were collected by filtration, washed with anhydrous acetone, and dried at 100 °C under vacuum overnight.

Optical pump-THz probe (OPTP) spectroscopy. In the OPTP measurement, the sample is first photoexcited by a 400 nm laser pulse (pulse duration 100 fs) to generate electrons and holes in the conduction and valence bands, respectively. Then, a single-cycle THz pulse with a pulse duration of 1 ps arrives, interacting with the photoexcited charge carriers, leaving a charge transport fingerprint in the transmitted THz pulse. The relative attenuation of the THz electric field $-\Delta E/E$ is proportional to the real part of the photoconductivity, while the phase shift is related to the imaginary part. The generation and recombination dynamics are obtained by changing the relative delay between the optical pulse and the THz pulse.

Doping experiment. All doping experiments were conducted in a nitrogen-filled glove box. The 2DPAV powder was drop-cast on the pre-patterned substrate. [4-(1,3-dimethyl-2,3-dihydro-1*H*-

benzoimidazol-2-yl)-phenyl]-dimethyl-amine (N-DMBI, dissolved in anhydrous acetonitrile) solution was coated on top of the 2DPAV powder and thermal-annealed at 150 °C to complete the doping process. Tetrakis(dimethylamino)ethylene (TDAE, 0.1-1 mL) and 2DPAV were tightly covered by a glass Petri dish at 50 °C.

Electrical conductivity measurements. Conductivity measurements were conducted using a two-probe method with a Keithley 2450 SourceMeter. The current-voltage (I-V) curve measurement was performed by sweeping the voltage in the range of −4.0 V to 4.0 V at room temperature.

A2. Calculation Method

To determine if the crystal structure is consistent with the experimental pXRD pattern, we employed a multi-level computational chemistry workflow that integrates PyMatgen, DFTB+, and VASP, supported by homemade scripts.[1] The electronic properties of the 2D PAVs were calculated using density functional theory (DFT) with the Vienna Ab Initio Simulation Package (VASP 5.4.4)[2], implementing the projector-augmented wave (PAW)[3] method and the Perdew-Burke-Ernzerhof (PBE)[4] functional. To account for London dispersion forces, Grimme's D3 correction was applied.[5] For lattice parameter optimization, a plane-wave cutoff energy of 400 eV was used, while a higher cutoff energy of 600 eV was employed for static calculations. The force convergence criterion during optimization was set to 0.02 eV $Å^{-1}$, and the energy convergence criterion in the self-consistent field iterations was 10^{-5} eV for optimization and 10^{-6} eV for static calculations. Partial charge density plots were generated using VESTA.[6] A k-point mesh of 2 × 2 × 4 was utilized for structural optimization, 2 × 2 × 8 for converged charge density calculations, and 5 × 5 × 10 for density of states (DOS) calculations. The frontier molecular orbitals and geometries are calculated using the DFT method with a basis set of B3LYP/6-31G(d,p). The relaxation times of electrons and holes in 2DPAV-TBDT-DPP-0 are estimated to be 1.97×10^{-13} and 1.52×10^{-14} s. The effective masses are obtained through parabolic fitting of the energy band curvature near the valence band maximum (VBM) and conduction band minimum (CBM), corresponding to holes and electrons, respectively.

Section B. Materials and Synthetic Procedures

General

Unless otherwise stated, all commercially available chemicals were used as received without further purification. The reactions were performed using standard vacuum-line and Schlenk techniques. Purification of all compounds was performed under air with reagent-grade solvents.

Synthesis

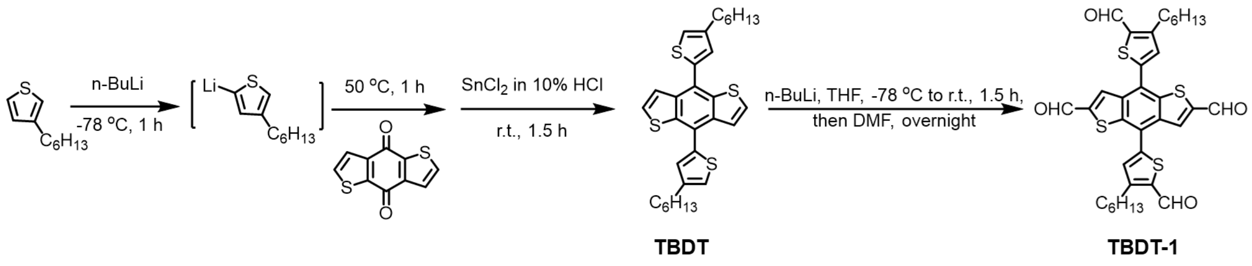

Synthetic route of monomers of TBDT-1.

Synthesis of 4,8-di(thiophen-2-yl)benzo[1,2-b:4,5-b']dithiophene (TBDT). TBDT was synthesized using an adapted method as described in a previous report.[7] ^{1}H NMR ($CDCl_3$, 300 MHz) δ 7.63 (d, J = 5.7 Hz, 2H), 7.46 (d, J = 5.6 Hz, 2H), 7.32 (d, J = 1.5 Hz, 2H), 7.11 (d, J = 1.4 Hz, 2H), 2.72 (t, J = 7.7 Hz, 4H), 1.72 (p, J = 7.4 Hz, 4H), 1.46–1.31 (m, 12H), 0.97–0.87 (m, 6H).

Synthesis of 4,8-bis(5-formyl-4-hexylthiophen-2-yl)benzo[1,2-*b*:4,5-*b*']dithiophene-2,6-dicarbaldehyde (TBDT-1). TBDT-1 was synthesized using an adapted method as described in a previous report.[7] ^{1}H NMR ($CDCl_3$, 300 MHz) δ 10.18 (s, 2H), 10.12 (s, 2H), 8.35 (s, 2H), 7.48 (s, 2H), 3.17–3.05 (m, 4H), 1.80 (t, J = 7.6 Hz, 4H), 1.47–1.34 (m, 12H), 0.95–0.88 (td, J = 7.1, 5.8, 3.5 Hz, 6H). ^{13}C NMR (CDCl3, 75 MHz) δ 184.32, 182.08, 153.32, 146.19, 145.29, 141.26, 139.33, 137.80, 132.69, 132.27, 127.13, 31.58, 31.53, 29.08, 28.71, 22.62, 14.09.

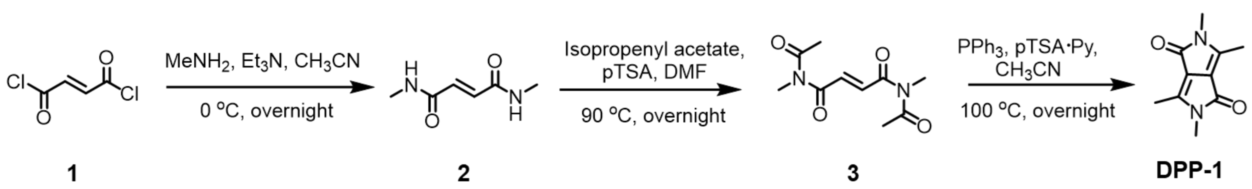

Synthetic route of DPP-1.

Synthesis of N^1,N^4-dihexylfumaramide (2). A 250 mL double-necked flask is connected with a pressure-equalizing dropping funnel and sealed. $MeNH_2$ (2.0 M in THF, 20 mL), Et_3N (3.7 mL), and acetonitrile (20 mL) were added with stirring and cooling to 0 °C. Fumaryl chloride (1.4 mL) in acetonitrile (20 mL) was then added dropwise through a dropping funnel during 1 hour at the same temperature. A white precipitate was observed as fumaryl chloride was added to the system. The reaction mixture was then kept stirring for 90 minutes at room temperature. After the reaction was completed, the solvent was removed under reduced pressure. The obtained residue was then partially dissolved and transferred into a 500 mL separating funnel with 300 mL of ethyl acetate. The organic layer was washed with a brine solution (4 * 100 mL). The organic layer was then evaporated under reduced pressure to obtain a white to brown solid, which was then partially dissolved with dichloromethane. The suspension solution was then transferred into 2 Petri dishes. After dichloromethane evaporated, the surface layer of brown solid was removed, and the lower layer of white solid was then collected and used for the next step.

Synthesis of N^1,N^4-diacetyl-N^1,N^4-dihexylfumaramide (3). 3.70 g of 2 was added to a 250 mL double-necked round-bottom flask with 200 mL of isopropenyl acetate and heated to 90 °C with stirring. 495.1 mg pTSA was dissolved in 10 mL of dimethylformamide (DMF) and then added to the bottle. The mixture was stirred and heated at 90 °C overnight. The mixture became a brown solution with some white precipitate. After cooling to room temperature, the mixture was concentrated under reduced pressure. The residue was dissolved in 180 mL ethyl acetate and washed with 1M HCl aqueous solution (2 * 100 mL), followed by saturated $NaHCO_3$ solution (2 * 100 mL) and water (100 mL). The organic phase was separated, dried over $MgSO_4$, and then concentrated under reduced pressure. The crude product was purified by column chromatography on silica gel with dichloromethane/ethyl acetate 6:1. The final product was collected as a white solid. ^{1}H NMR ($CDCl_3$, 300 MHz) δ 7.42 (s, 1H), 3.29 (s, 3H), 2.43 (s, 3H).

Synthesis of 2,5-dihexyl-3,6-dimethyl-2,5-dihydropyrrolo[3,4-*c*]pyrrole-1,4-dione (DPP-1). 1.13 g of 3, 1.31 g of PPh3, and 628.3 mg of pyridinium *p*-toluenesulfonate were added to a 100 mL round-bottom flask containing a magnetic stir bar in 25 mL of acetonitrile. The reaction was stirred at 100 °C overnight. After cooling to room temperature, the mixture was concentrated under reduced pressure. The residue was dissolved in 100 mL ethyl acetate and washed with water (4 * 100 mL). The organic phase was separated, dried over $MgSO_4$, filtered, and concentrated under reduced pressure. The crude product was purified by column chromatography on silica gel using

a gradient of dichloromethane/ethyl acetate 50:1 to 20:1. The collected product was then concentrated under reduced pressure and recrystallized with isohexane. ^{1}H NMR ($CDCl_3$, 300 MHz) δ 3.09 (s, 6H), 2.33 (s, 6H). ^{13}C NMR ($CDCl_3$, 76 MHz) δ 162.18, 146.16, 108.25, 26.19, 12.86. M.P. ca. 240 °C.

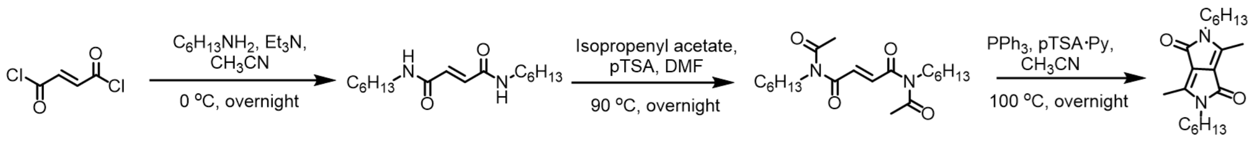

Synthetic route of (DPP-2).

Synthesis of *N*¹,*N*⁴-dihexylfumaramide (4). A 250 mL double-necked flask is connected with a pressure-equalizing dropping funnel and sealed. Hexylamine (5.2 mL), Et_3N (3.6 mL), and acetonitrile (2 * 20 mL) were added with stirring and cooled to 0 °C. Fumaryl chloride (1.4 mL) and acetonitrile (20 mL) were then added dropwise through a dropping funnel during 50 minutes at the same temperature. A white precipitate was observed as fumaryl chloride was added to the system. The reaction mixture was then kept stirring for 90 minutes at room temperature. After the reaction was completed, the solvent was removed under reduced pressure. The obtained residue was then partially dissolved and transferred into a 500 mL separating funnel with 300 mL of ethyl acetate. The organic layer was washed with brine solution (4 * 100 mL). The organic layer was then evaporated under reduced pressure to obtain a white to brown solid, which was then partially dissolved with dichloromethane. The suspension solution was then transferred into 2 Petri dishes. After dichloromethane evaporated, the surface layer of brown solid was removed, and the lower layer of white solid was then collected. 2.58 g of product was finally collected. ^{1}H NMR (DMSO, 500 MHz) δ 8.33 (t, J = 5.7 Hz, 2H), 6.76 (s, 2H), 3.15–3.04 (m, 4H), 1.4–1.35 (m, 4H), 1.26–1.20 (m, 12H), 0.86–0.81 (m, 6H).

Synthesis of *N*¹,*N*⁴-diacetyl-*N*¹,*N*⁴-dihexylfumaramide (5). 2.58 g **4** was added to a 250 mL double-necked round bottle containing 100 mL isopropenyl acetate and heated to 90 °C with stirring. 197.6 mg pTSA was dissolved in 10 mL DMF and then added to the bottle. The mixture was stirred and heated at 90 °C overnight. The mixture became a brown solution with some white precipitate. After cooling to room temperature, the mixture was concentrated under reduced pressure. The residue was dissolved in 180 mL ethyl acetate and washed with 1M HCl aqueous

solution (2 * 100 mL), followed by saturated $NaHCO_3$ solution (2 * 100 mL) and water (100 mL). The organic phase was separated, dried over $MgSO_4$, and concentrated under reduced pressure. The crude product was purified by column chromatography on silica gel using a 20:1 mixture of dichloromethane and ethyl acetate. The final product was a yellow-brown oil. ^{1}H NMR ($CDCl_3$, 500 MHz) δ 7.35 (s, 2H), 3.74–3.65 (m, 4H), 2.42 (s, 6H), 1.58 (s, 4H), 1.30 (dd, J = 3.7, 1.8 Hz, 12H), 0.87 (d, J = 6.9 Hz, 6H).

Synthesis of 2,5-dihexyl-3,6-dimethyl-2,5-dihydropyrrolo[3,4-*c*]pyrrole-1,4-dione (DPP-2). 1.8 g of 5, 1.29 g of PPh_3, and 619 mg of pyridinium *p*-toluenesulfonate were added to a 100 mL round-bottom flask with a magnetic stir bar in 25 mL of acetonitrile. The reaction was stirred at 100 °C overnight. After cooling to room temperature, the mixture was concentrated under reduced pressure. The residue was dissolved in 100 mL of ethyl acetate and washed with water (4 * 100 mL). The organic phase was separated, dried over magnesium sulfate ($MgSO_4$), filtered, and concentrated under reduced pressure. The crude product was purified by column chromatography on silica gel using a gradient of dichloromethane/ethyl acetate from 50:1 to 20:1. The collected product was then concentrated under reduced pressure and recrystallized with isohexane. ^{1}H NMR ($CDCl_3$, 300 MHz) δ 3.55–3.45 (m, 4H), 2.33 (s, 6H), 1.64–1.47 (m, 4H), 1.40–1.19 (m, 13H), 0.93–0.81 (m, 6H). ^{13}C NMR ($CDCl_3$, 75 MHz) δ 162.24, 145.89, 108.46, 77.47, 77.25, 77.05, 76.63, 40.15, 31.49, 29.76, 26.53, 22.54, 14.03, 12.77. M.P. ca. 86 °C.

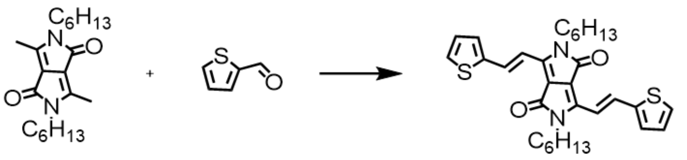

Solution synthesis of 2,5-dihexyl-3,6-bis((*E*)-2-(thiophen-2-yl)vinyl)-2,5-dihydropyrrolo[3,4-*c*]pyrrole-1,4-dione (Model compound 1). DPP-2 (20 mg, 0.06 mmol), thiophene-2-carbaldehyde (Th-CHO) (14.84 mg, 0.132 mmol), and sodium benzoate (1 mg) were added into a Synthware thick wall pressure bottle fitted with toluene/methanol (5:1) under argon protection. Continue stirring at 120 °C overnight, then cool to room temperature. The crude product was separated by column chromatography on silica gel using a gradient of dichloromethane/hexane from 50:1 to 20:1. The model compound was collected as a purple solid in 65% yield. ^{1}H NMR ($CDCl_3$, 300 MHz) δ 9.03 (d, J = 15.5 Hz, 2H), 7.39 (dd, J = 6.6, 4.4 Hz, 4H), 7.09 (dd, J = 5.0, 3.7 Hz, 2H), 6.63 (d, J = 15.5 Hz, 2H), 3.79 (t, J = 7.5 Hz, 4H), 1.67 (q, J = 7.3 Hz, 4H), 1.35 (d,

J = 2.4 Hz, 12H), 0.90 (s, 6H). ^{13}C NMR ($CDCl_3$, 76 MHz) δ 161.42, 144.02, 142.43, 135.82, 131.16, 128.74, 128.46, 112.99, 109.50, 40.74, 31.60, 30.10, 26.67, 22.67, 14.17.

Synthesis of 2DPAV-TBDT-DPP-1. A 5 mL high-pressure glass tube was charged with TBDT-1 (30.00 mg, 0.047 mmol), DPP-1 (18.17 mg, 0.095 mmol), and sodium benzoate (27.24 mg, 0.189 mmol). The tube was sonicated at room temperature for 3 min, degassed by three freeze-pump-thaw cycles, sealed under vacuum, and heated at 200 °C for three days. After cooling to room temperature, the precipitate was filtered, washed with dimethylformamide, acetone, water, tetrahydrofuran, and anhydrous acetone, respectively. It was then collected and dried under vacuum at 100 °C overnight to yield a dark powder in 94% yield.

Synthesis of 2DPAV-TBDT-DPP-2. A 5 mL high-pressure glass tube was charged with TBDT-1 (30.00 mg, 0.047 mmol), DPP-2 (31.42 mg, 0.095 mmol), and sodium benzoate (27.24 mg, 0.189 mmol). The tube was sonicated at room temperature for 3 min, degassed by three freeze-pump-thaw cycles, sealed under vacuum, and heated at 200 °C for three days. After cooling to room temperature, the precipitate was filtered, washed with dimethylformamide, acetone, water, tetrahydrofuran, and anhydrous acetone, respectively. It was then collected and dried under vacuum at 100 °C overnight to yield a dark powder in 96% yield.

Section C. Supporting Figures

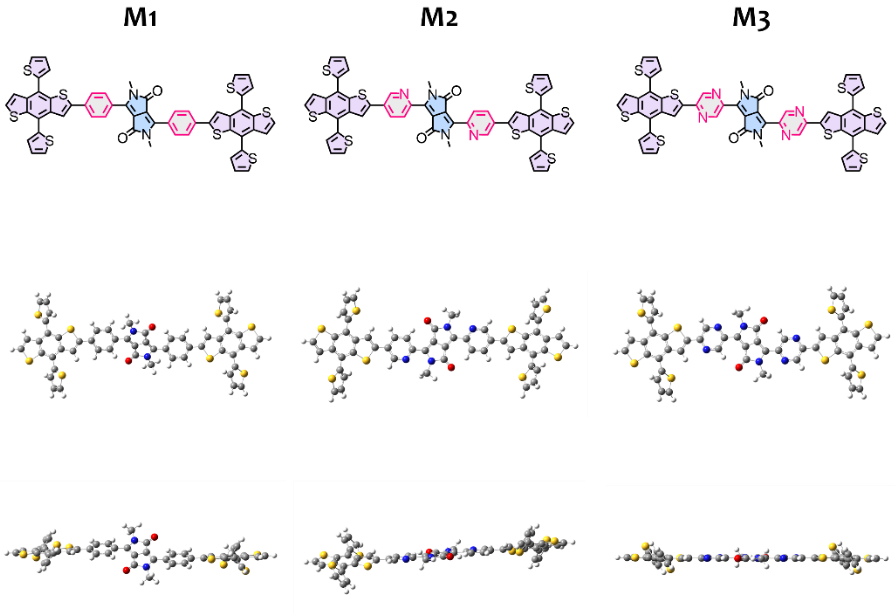

Supplementary Figure 1. Optimized geometries of model compounds with the six-membered ring as the bridge between TBDT and DPP unit. Top: chemical structures (top), top-view (middle), and side-view (bottom) of M1, M2, and M3.

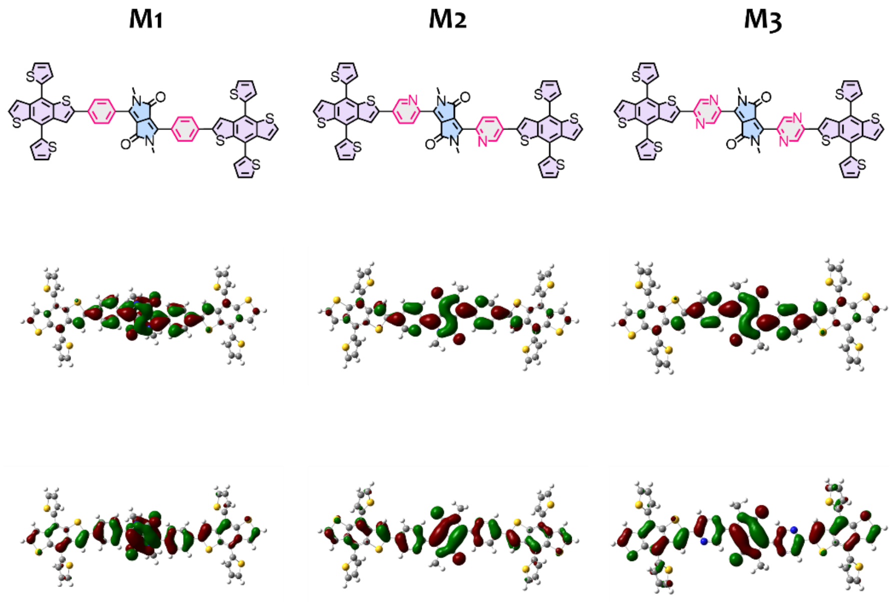

Supplementary Figure 2. Optimized Kohn-Sham molecular orbitals of model compounds with the six-membered ring as the bridge between TBDT and DPP unit. Top: chemical structures (top), LUMO orbitals (middle), and HOMO orbitals (bottom) of M1, M2, and M3.

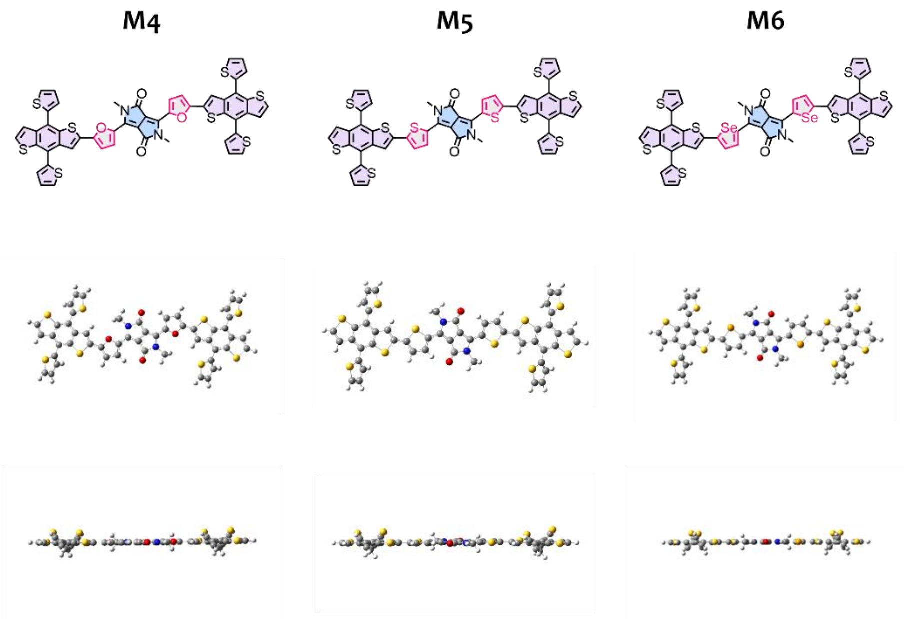

Supplementary Figure 3. Optimized geometries of model compounds with the five-membered ring as the bridge between TBDT and DPP unit. Top: chemical structures (top), LUMO orbitals (middle), and HOMO orbitals (bottom) of M4, M5, and M6.

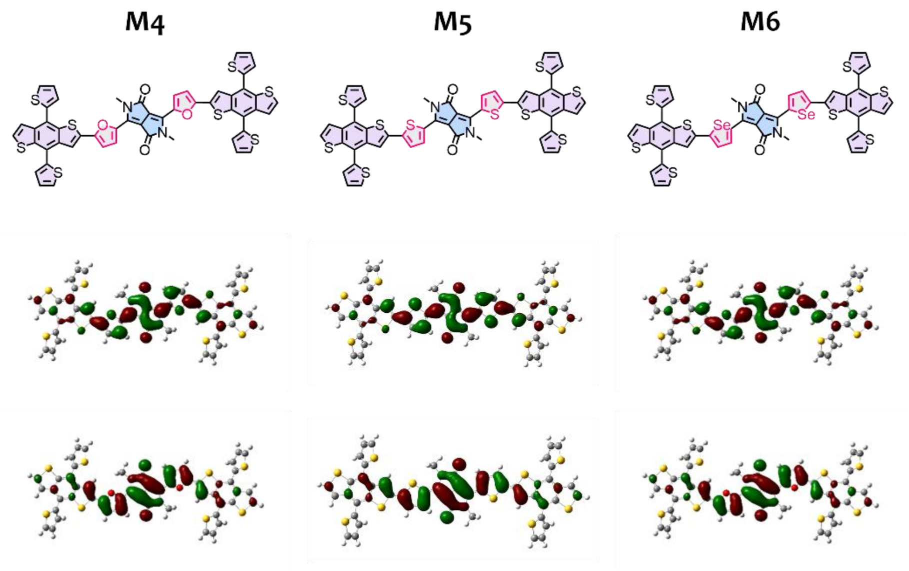

Supplementary Figure 4. Optimized Kohn-Sham molecular orbitals of model compounds with the five-membered ring as the bridge between TBDT and DPP unit. Top: chemical structures (top), LUMO orbitals (middle), and HOMO orbitals (bottom) of M4, M5, and M6.

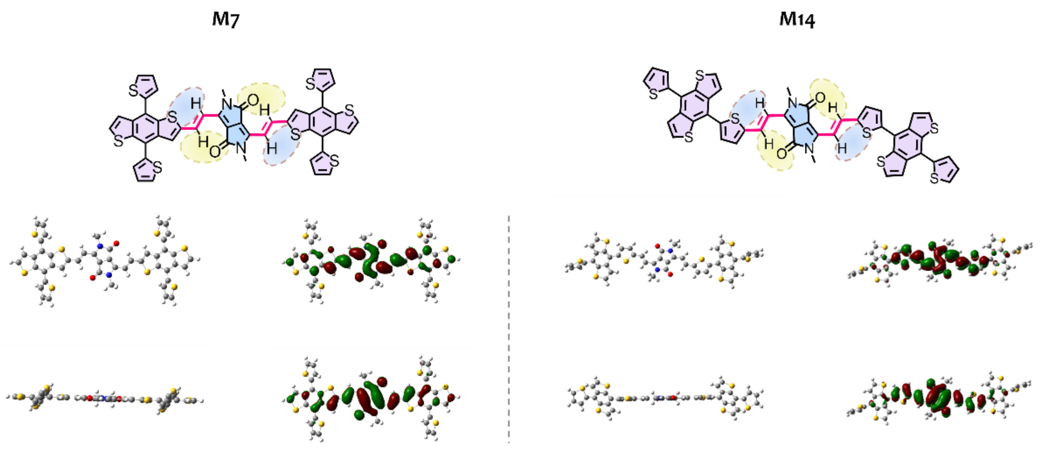

Supplementary Figure 5. Optimized geometries and Kohn-Sham molecular orbitals of the model compound with the vinylene linkage between TBDT and DPP unit.

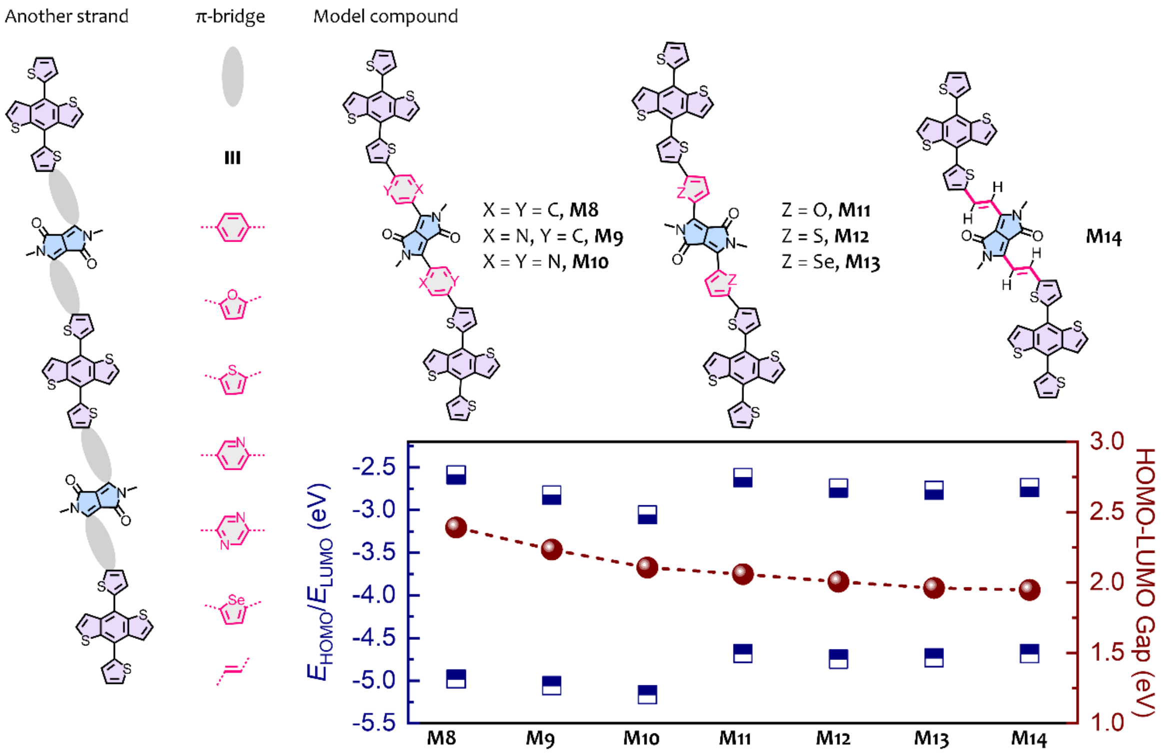

Supplementary Figure 6. Structure of strand-y of the 2DPAV-TBDT-DPP. Chemical structures of model compounds in another direction and the HOMO/LUMO energy alignment and the HOMO-LUMO gap.

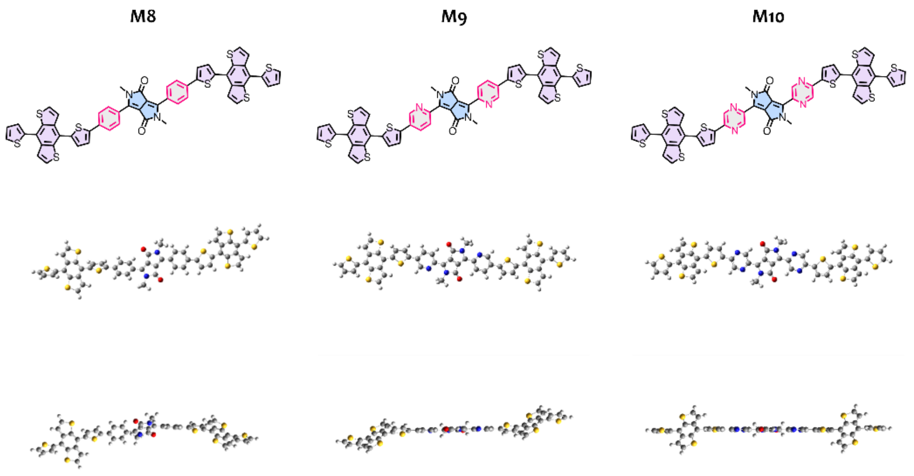

Supplementary Figure 7. Optimized geometries of model compounds with the six-membered ring as the bridge between TBDT and DPP unit. Top: chemical structures (top), top-view (middle), and side-view (bottom) of M8, M9, and M10.

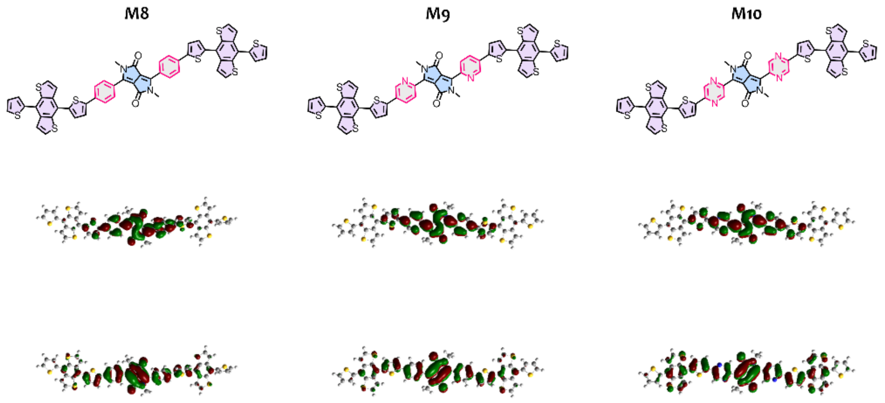

Supplementary Figure 8. Optimized Kohn-Sham molecular orbitals of model compounds with the six-membered ring as the bridge between TBDT and DPP unit. Top: chemical structures (top), LUMO orbitals (middle), and HOMO orbitals (bottom) of M8, M9, and M10.

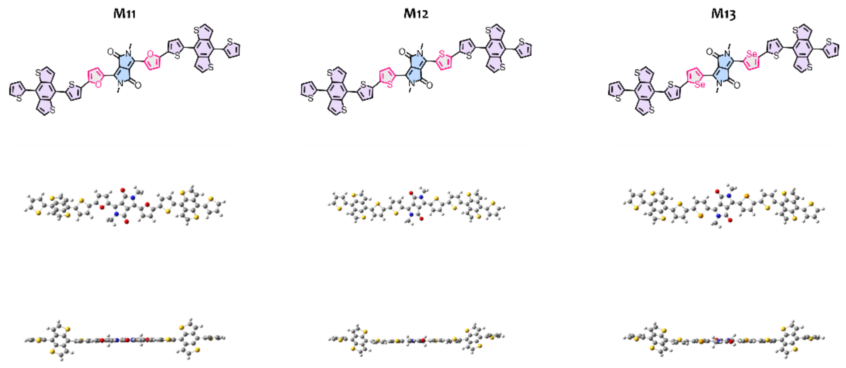

Supplementary Figure 9. Optimized geometries of model compounds with the five-membered ring as the bridge between TBDT and DPP unit. Top: chemical structures (top), top-view (middle), and side-view (bottom) of M11, M12, and M13.

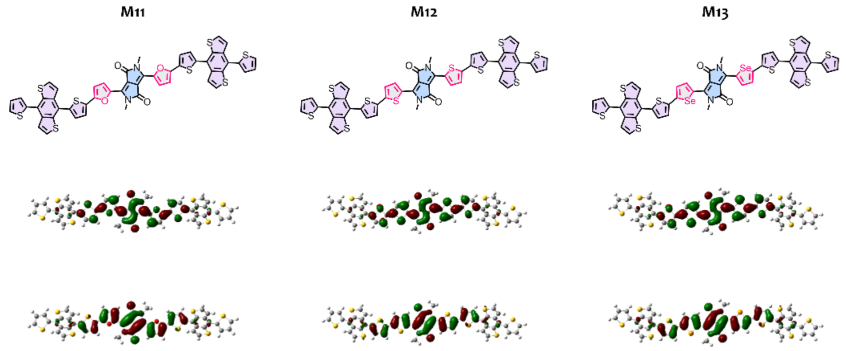

Supplementary Figure 10. Optimized Kohn-Sham molecular orbitals of model compounds with the five-membered ring as the bridge between TBDT and DPP unit. Top: chemical structures (top), LUMO orbitals (middle), and HOMO orbitals (bottom) of M11, M12, and M13.

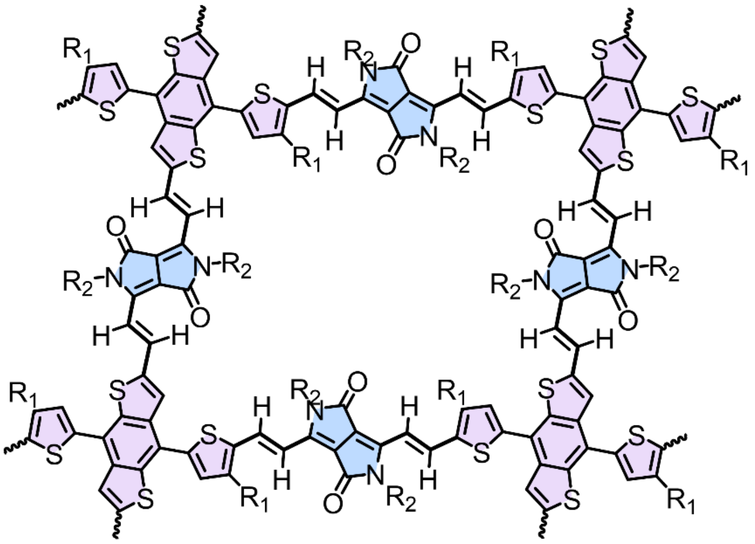

$R_1 = R_2$ = Methyl, **2DPAV-TBDT-DPP-0**
R_1 = Hexyl, R_2 = Methyl, **2DPAV-TBDT-DPP-1**
$R_1 = R_2$ = Hexyl, **2DPAV-TBDT-DPP-2**

Supplementary Figure 11. Chemical structures of the 2D PAVs discussed in this work.

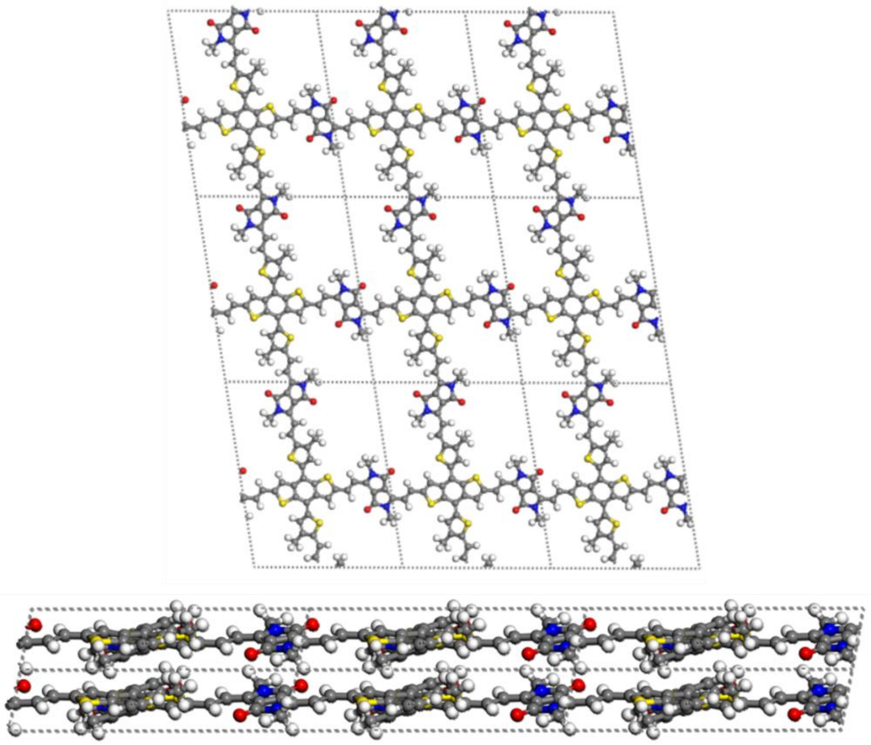

Supplementary Figure 12. Models of 2DPAV-TBDT-DPP-0.

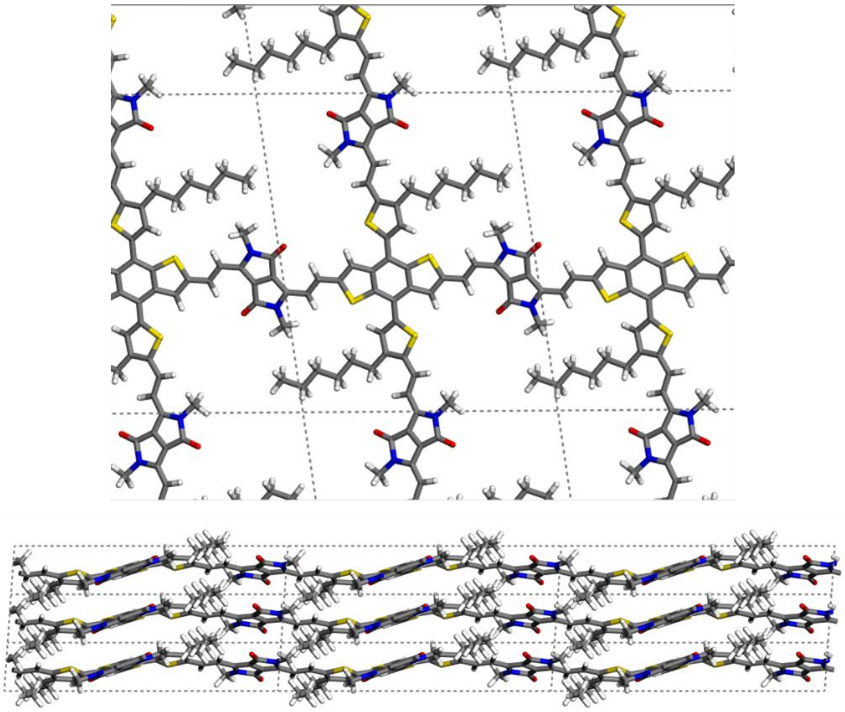

Supplementary Figure 13. Models of 2DPAV-TBDT-DPP-1.

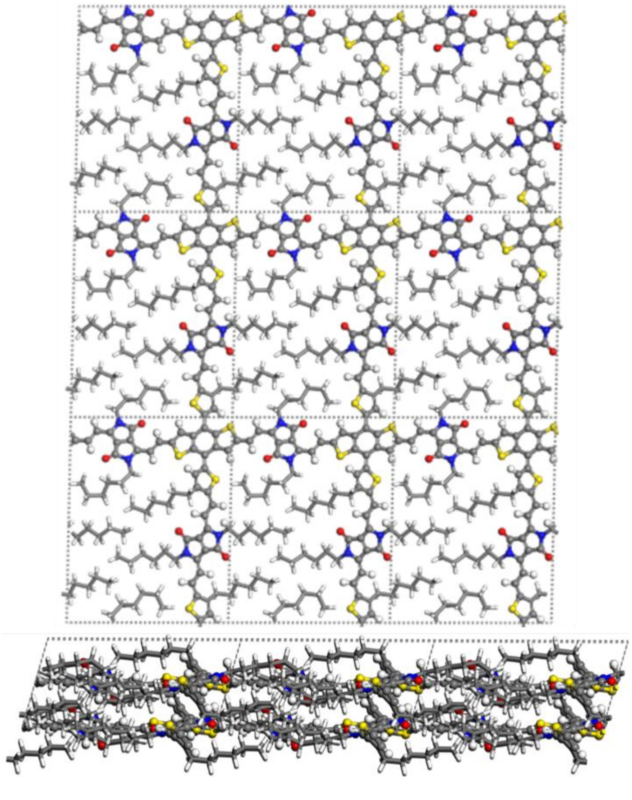

Supplementary Figure 14. Models of 2DPAV-TBDT-DPP-2.

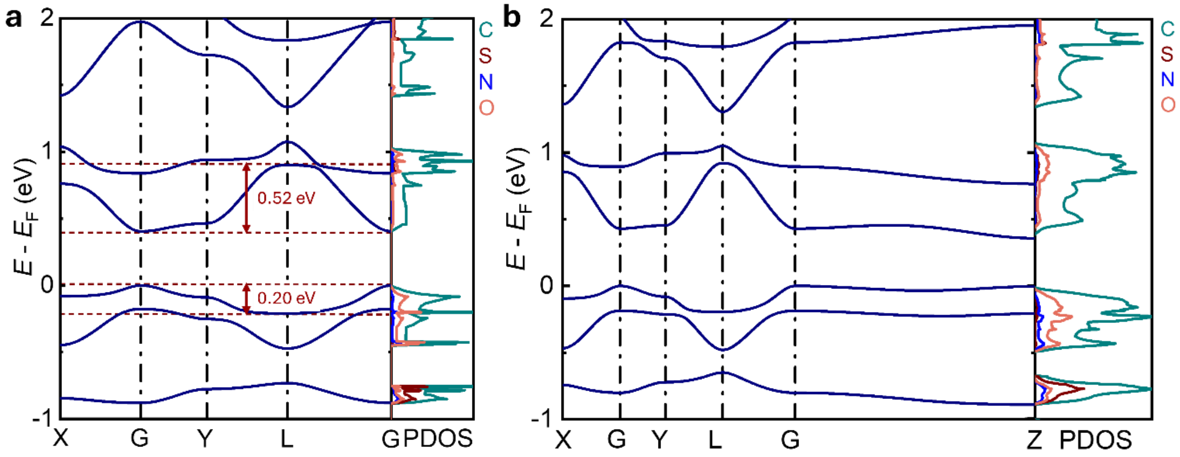

Supplementary Figure 15. Electronic band structures and PDOS (C*p*, N*p*, O*p,* and S*p*) of monolayer (left) and layer-stacked (right) 2DPAV-TBDT-DPP-2, respectively.

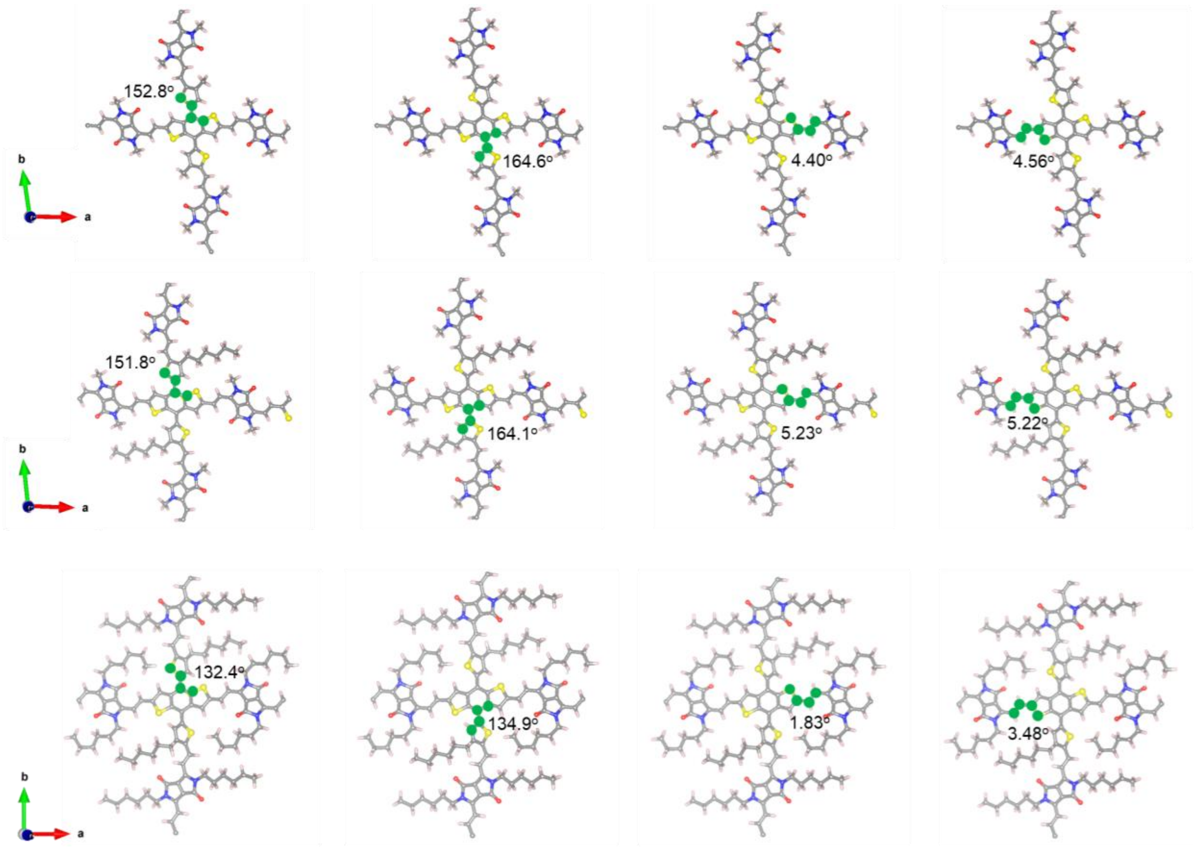

Supplementary Figure 16. Analysis of the torsion angles of optimized models of 2DPAV-TBDT-DPP-0 (top), 2DPAV-TBDT-DPP-1 (middle), and 2DPAV-TBDT-DPP-2 (bottom).

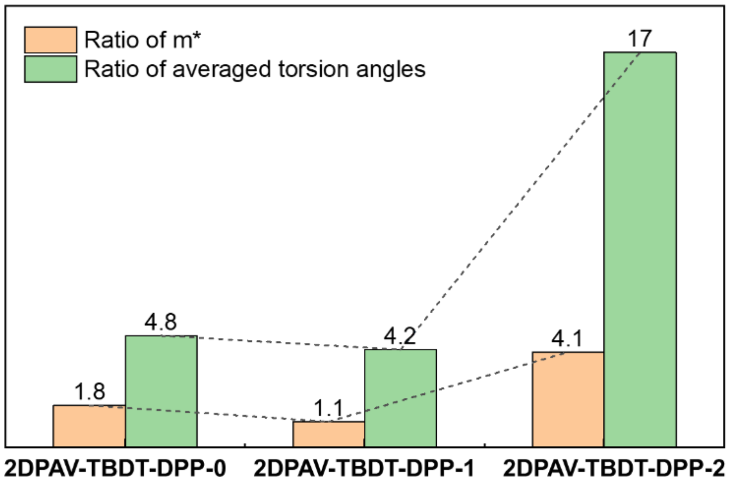

Supplementary Figure 17. Analysis of the ratio of effective masses and the ratio of averaged torsion angles along *a* and *b* directions.

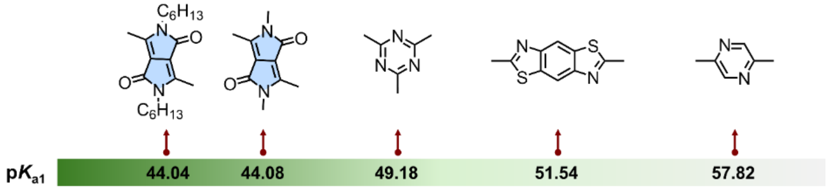

Supplementary Figure 18. Derived pK_{a1} values of different electron-deficient units. The pK_{a1} values are calculated according to the previous report.[8] Smaller pK_{a1} values indicate a high reactivity of the methyl groups upon deprotonation to give carbanion intermediates. The results show almost identical pK_{a1} values of ~44 for DPP-1 and DPP-2, which are much lower than that of 58 for 2,5-dimethylpyrazine, indicative of the higher reactivity, indicating superior deprotonation ability of methyl groups in the two DPP monomers to generate carbanions and trigger the condensation reaction.

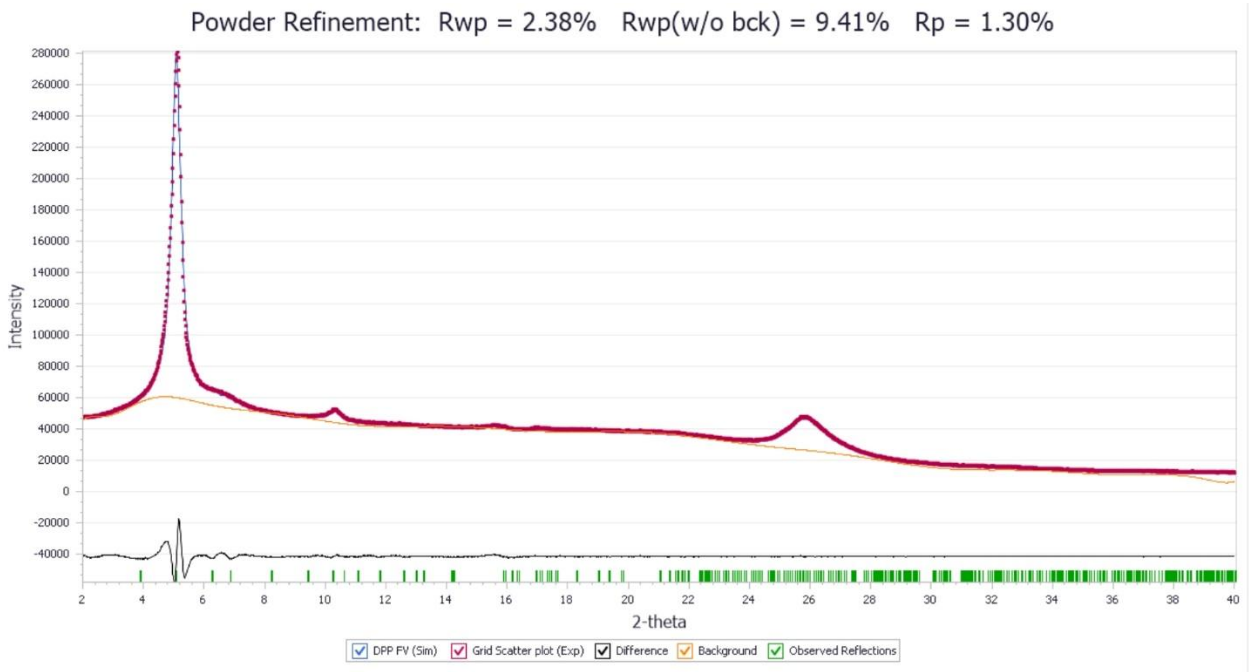

Supplementary Figure 19. Pawley refinement of 2DPAV-TBDT-DPP-1.

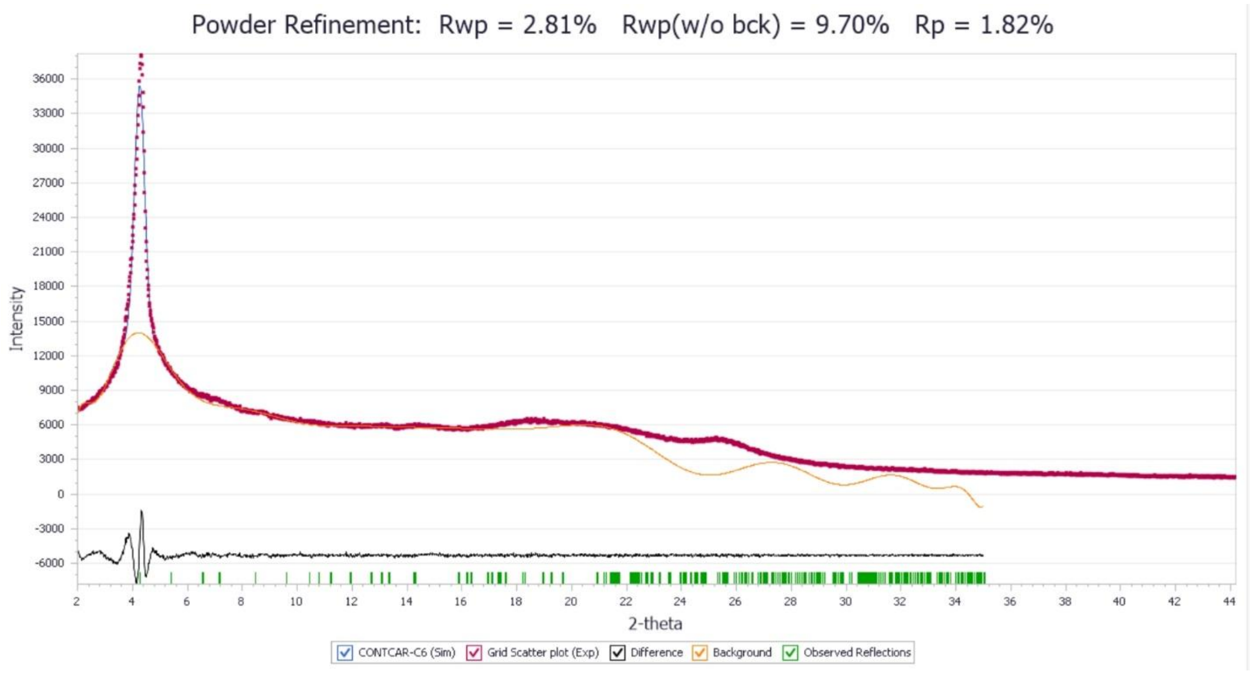

Supplementary Figure 20. Pawley refinement of 2DPAV-TBDT-DPP-2.

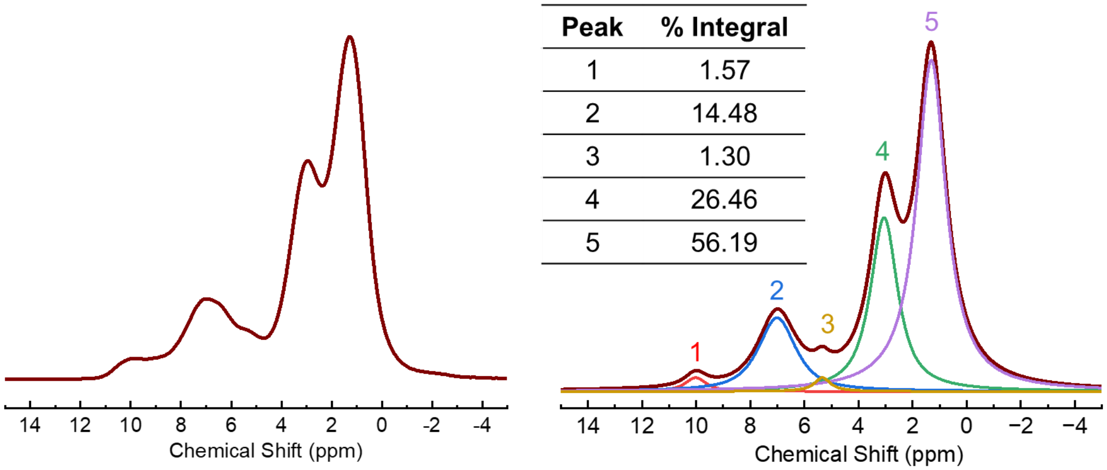

Peak	% Integral
1	1.57
2	14.48
3	1.30
4	26.46
5	56.19

Supplementary Figure 21. Measured ss-depth ^{1}H NMR spectrum (left) of 2DPAV-TBDT-DPP-1 and deconvolution[9] (right) of the spectrum. The ratio of the unreacted aldehyde C is determined to be 1.57%. There are 2 unreacted aldehyde groups remaining per ~ 3 BDT nodes in the lattice. Such structural imperfections are a major factor contributing to the inferior long-range charge transport in COFs relative to structurally similar linear conjugated polymers.

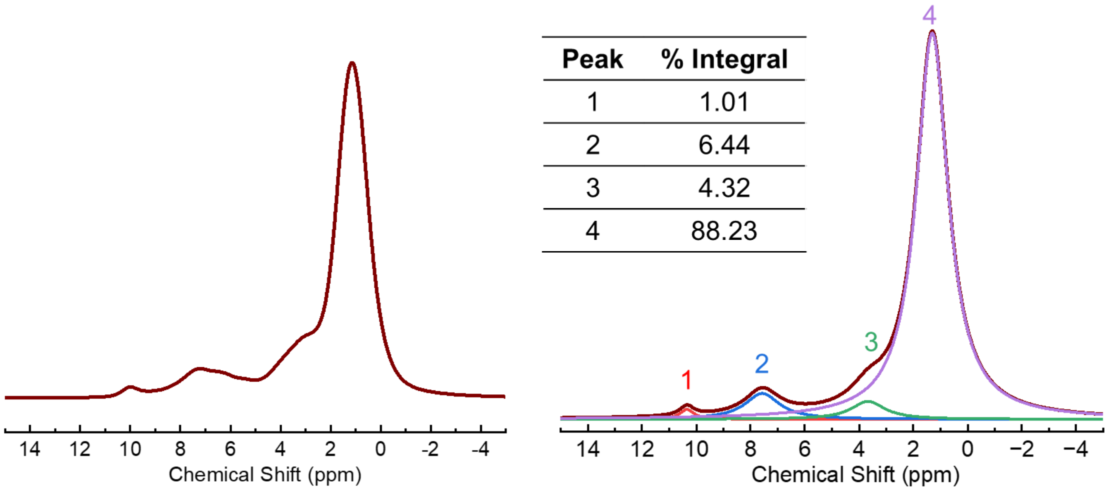

Peak	% Integral
1	1.01
2	6.44
3	4.32
4	88.23

Supplementary Figure 22. Measured ss-depth ^{1}H NMR spectrum (left) of 2DPAV-TBDT-DPP-2 and deconvolution[9] (right) of the spectrum. The ratio (f) of the unreacted aldehyde C is determined to be 1.01%. For a rhombic lattice, the number of edge sites scales linearly with the domain size (L), whereas the total number of repeat units scales with L^2, giving the relationship $f = 4/L$, we obtain $L \approx 396$ repeat units per edge and thus approximately 1.6×10^5 repeat units within a single 2D domain. Multiplying by the repeat-unit molecular weight (1215.79 g/mol) gives an estimated domain molecular weight of ~ 1.9×10^8 g/mol (~190,000 kDa).

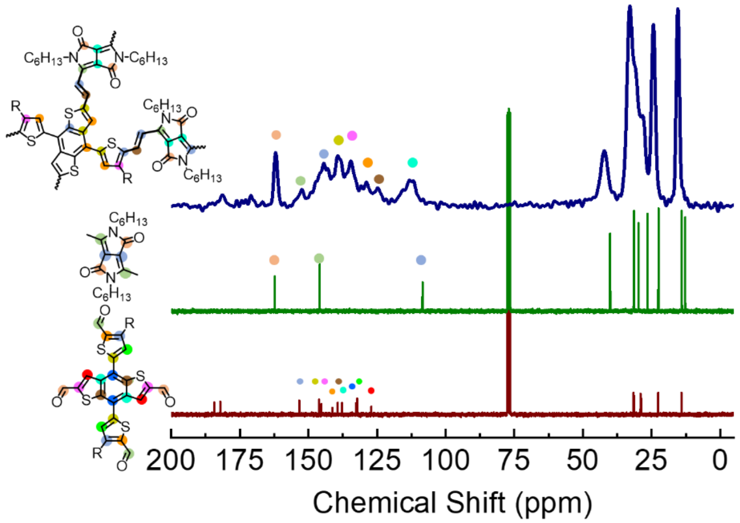

Supplementary Figure 23. ^{13}C CP MAS NMR spectra of 2DPAV-TBDT-DPP-2.

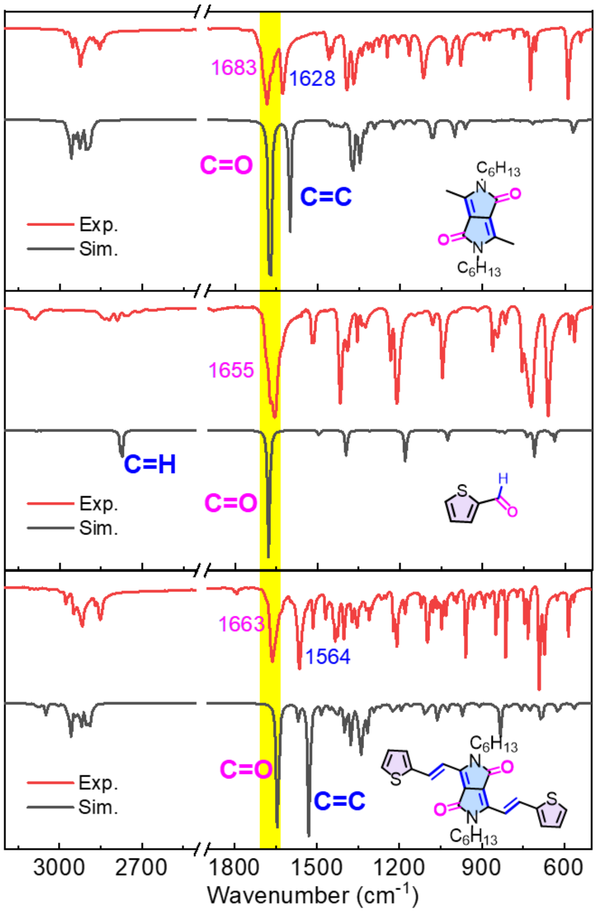

Supplementary Figure 24. Experimental (Exp.) and simulated (Sim.) FT-IR spectra of DPP-2 (top), Th-CHO (middle), and Model compound 1 (bottom).

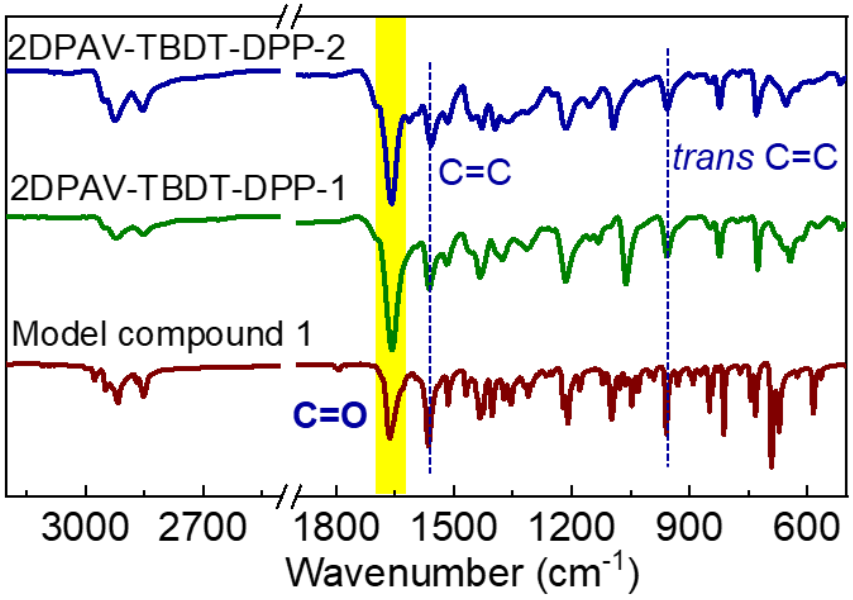

Supplementary Figure 25. FT-IR spectra of model compound 1, 2DPAV-TBDT-DPP-1, and 2DPAV-TBDT-DPP-2.

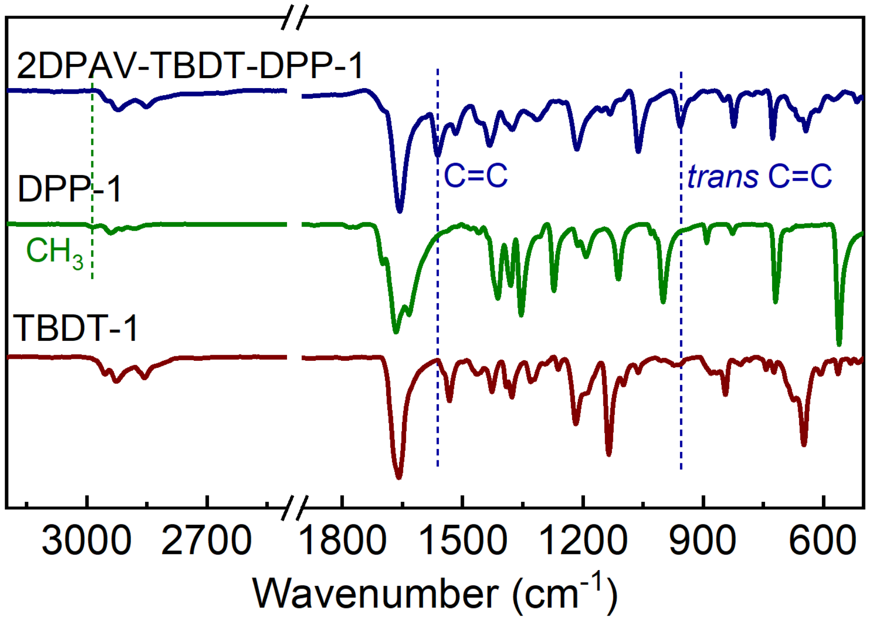

Supplementary Figure 26. FT-IR spectra of 2DPAV-TBDT-DPP-1.

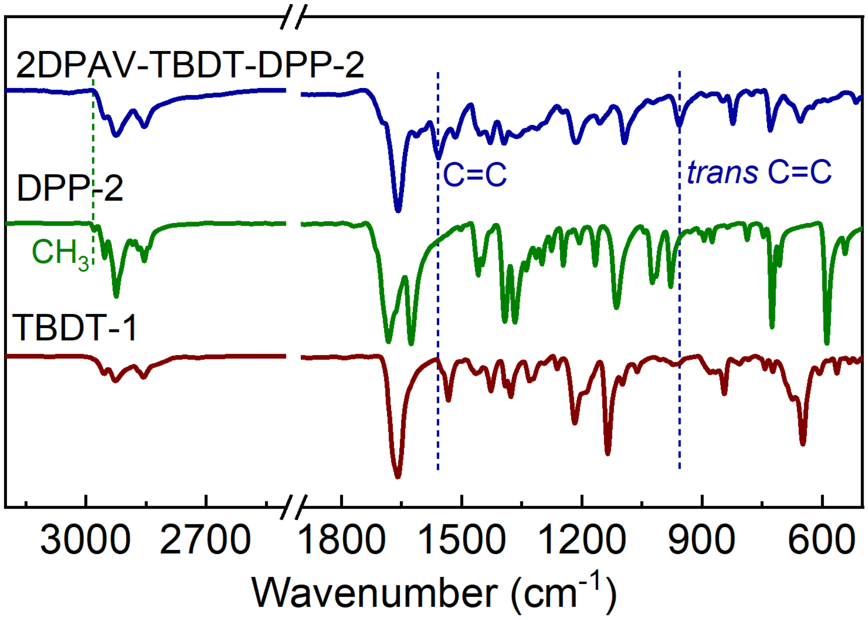

Supplementary Figure 27. FT-IR spectra of 2DPAV-TBDT-DPP-2.

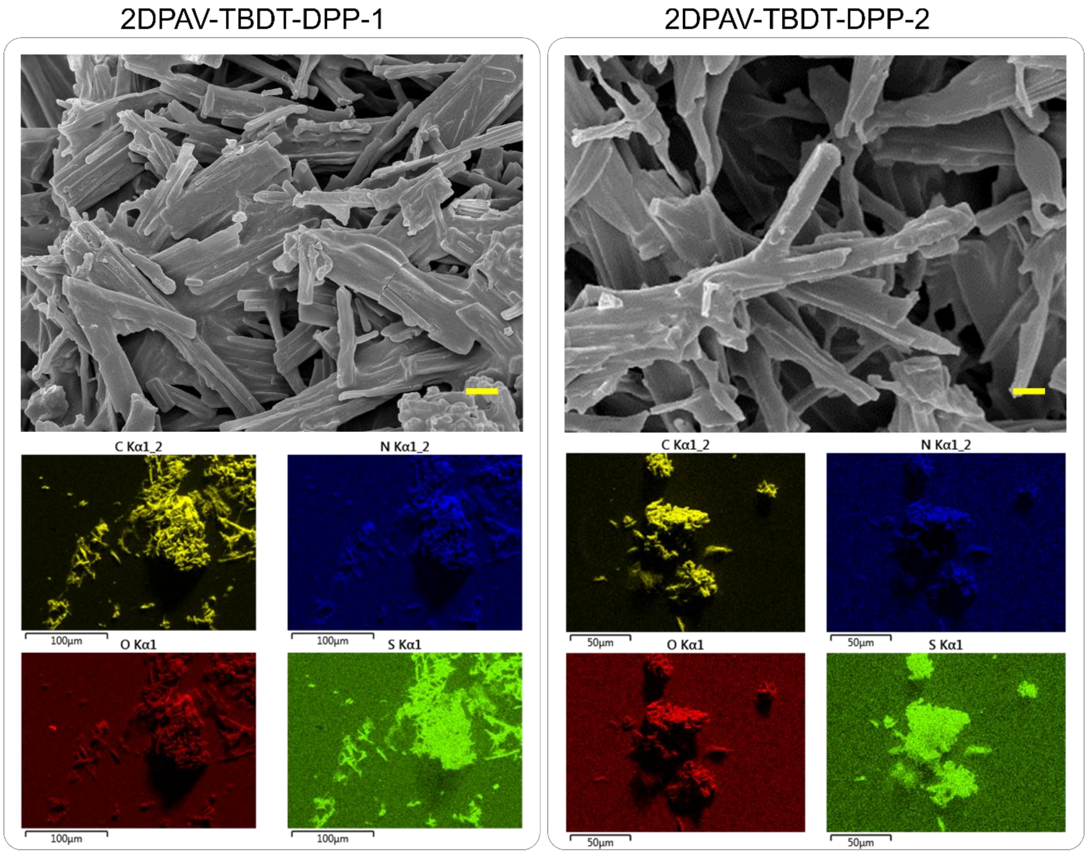

Supplementary Figure 28. SEM images (the scale bar represents 1 μm) and EDS mapping.

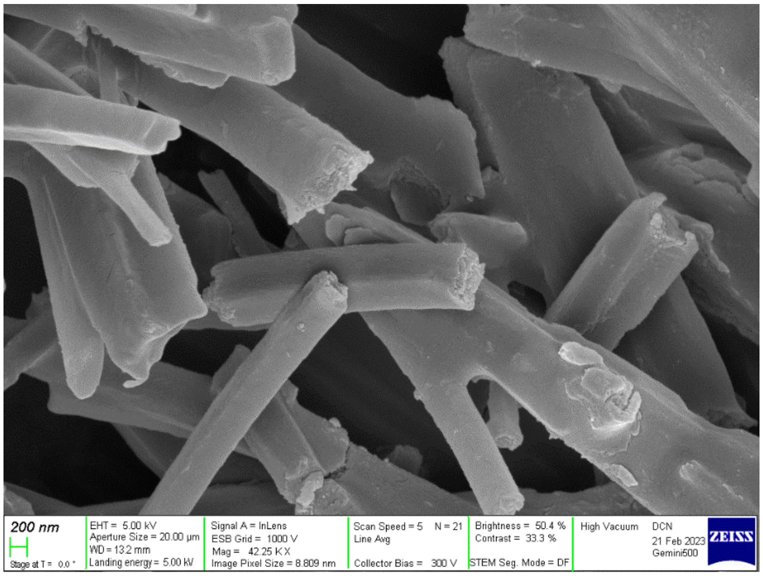

Supplementary Figure 29. Representative SEM image for 2DPAV-TBDT-DPP-2.

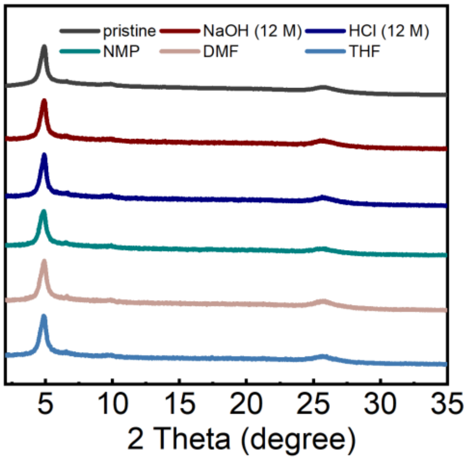

Supplementary Figure 30. pXRD patterns before and after the chemical stability study.

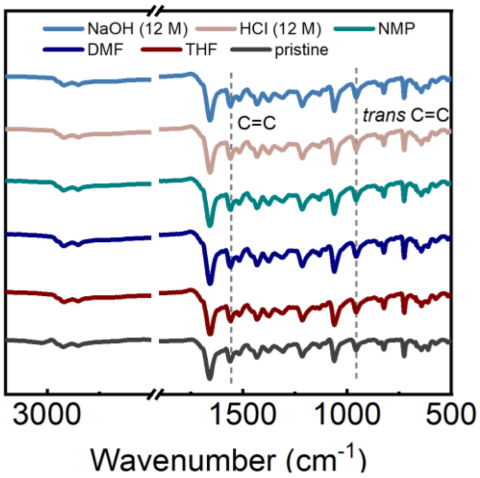

Supplementary Figure 31. FT-IR spectra before and after the chemical stability study.

The chemical stability of 2D PAVs was evaluated by dispersing the powders in various solvents, including tetrahydrofuran (THF), dimethylformamide (DMF), *N*-methylpyrrolidone (NMP), 12 M HCl, and 12 M NaOH at room temperature for 24 hours. After filtration and drying under vacuum, the undetectable deviations in the FT-IR spectra and pXRD patterns indicate superb structure stability of both 2D PAVs. Thermogravimetric analysis reveals that both 2D PAVs are thermally stable up to approximately 400 °C.

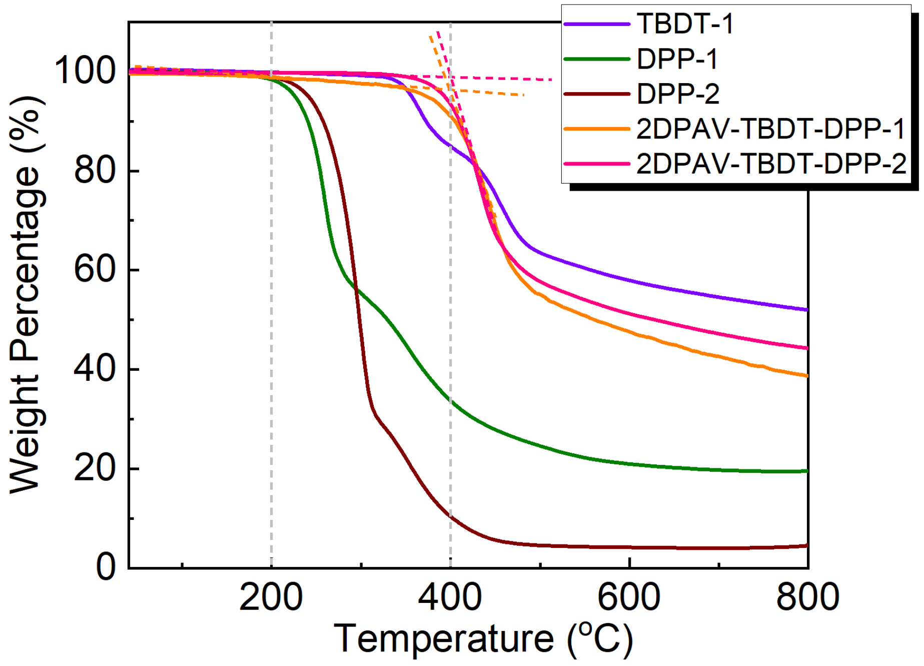

Supplementary Figure 32. Thermogravimetric analysis of the monomers and the 2D PAVs.

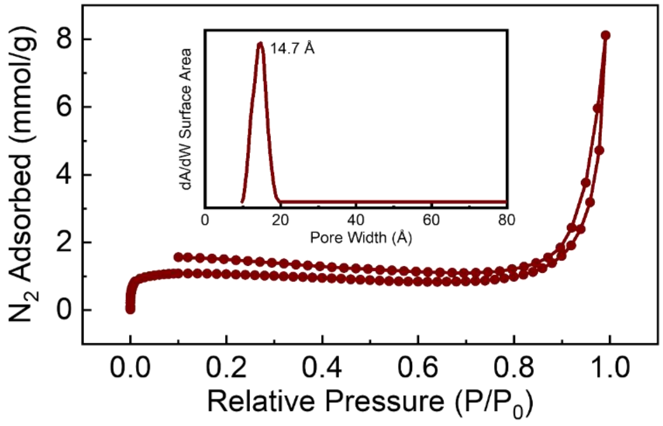

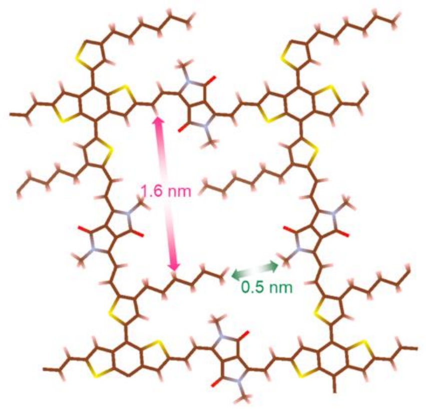

Supplementary Figure 33. Nitrogen physisorption and pore size distribution of 2DPAV-TBDT-DPP-1 and its structural model.

The Brunauer–Emmett–Teller (BET) surface area of 2DPAV-TBDT-DPP-1 was estimated to be about 123 m^2 g^{-1} (Supplementary Figure 17). The main pore size was determined as 1.47 nm using the nonlocal DFT method, which falls between the smallest (0.5 nm) and the largest (1.6 nm) wall-to-wall distances of the framework.

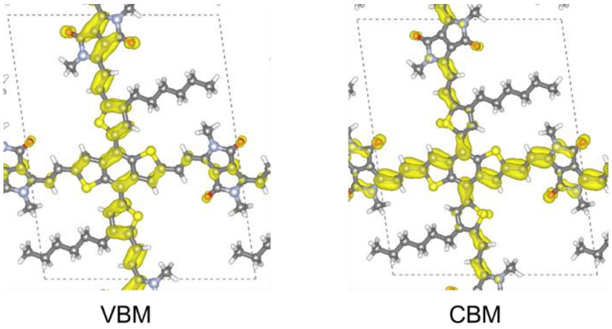

Supplementary Figure 34. The partial (band decomposed) charge density of the VBM and CBM of monolayered 2DPAV-TBDT-DPP-1.

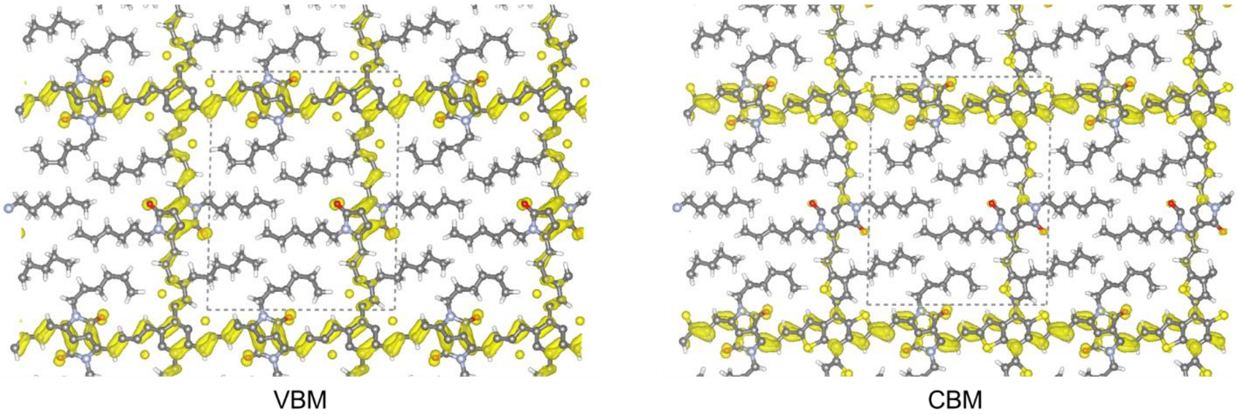

Supplementary Figure 35. The partial (band decomposed) charge density of the VBM and CBM of monolayered 2DPAV-TBDT-DPP-2.

For 2DPAV-TBDT-DPP-1, CBM and VBM delocalize along the two different directions, that is, in accordance with the different preferred directions of electrons and holes (preferred direction shows more dispersive band structure, thus a smaller effective mass, for electrons, along a(x), for holes, along b(y)). For 2DPAV-TBDT-DPP-2, both CBM and VBM electron clouds predominantly delocalize along one direction. In contrast, in another direction, they show limited electron distribution owing to the twisted configuration caused by the steric hindrance of the alkyl side chains.

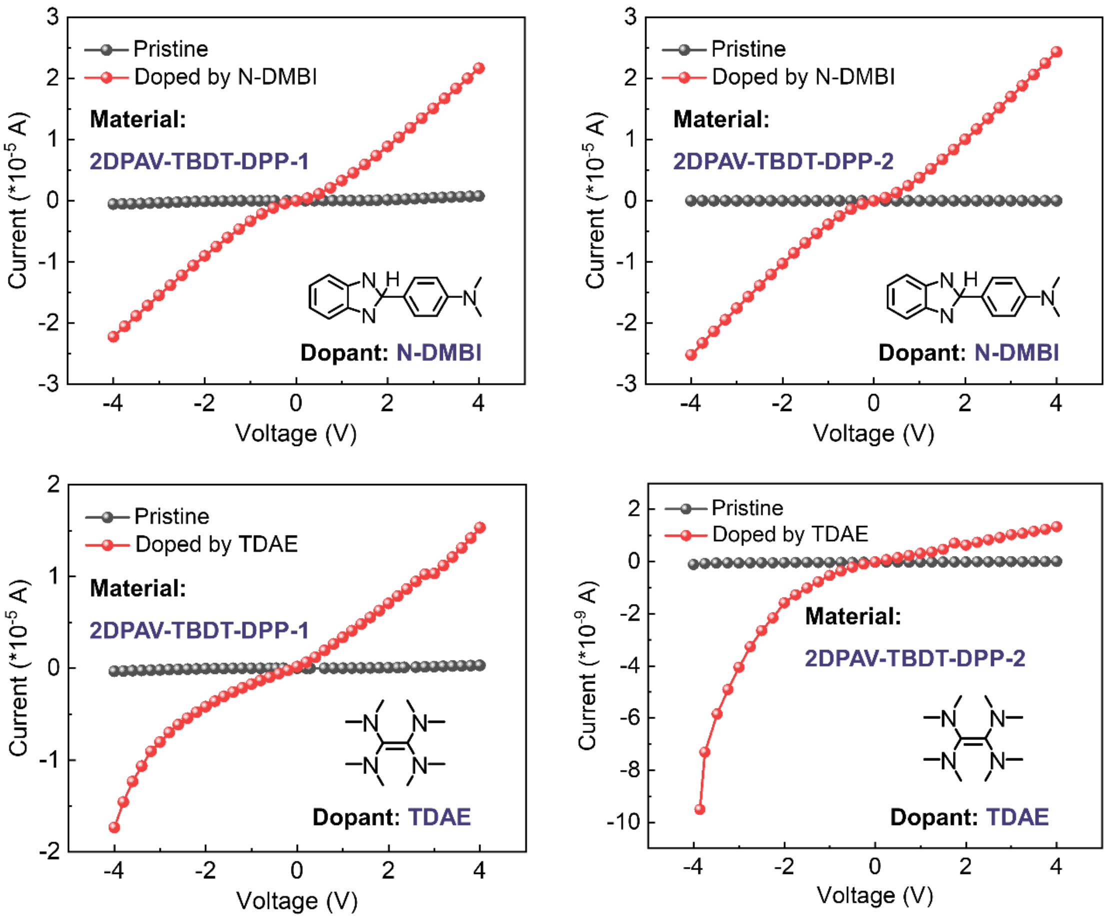

Supplementary Figure 36. I-V curves of 2DPAV-TBDT-DPP-1/2 by *N*-DMBI and TDAE doping.

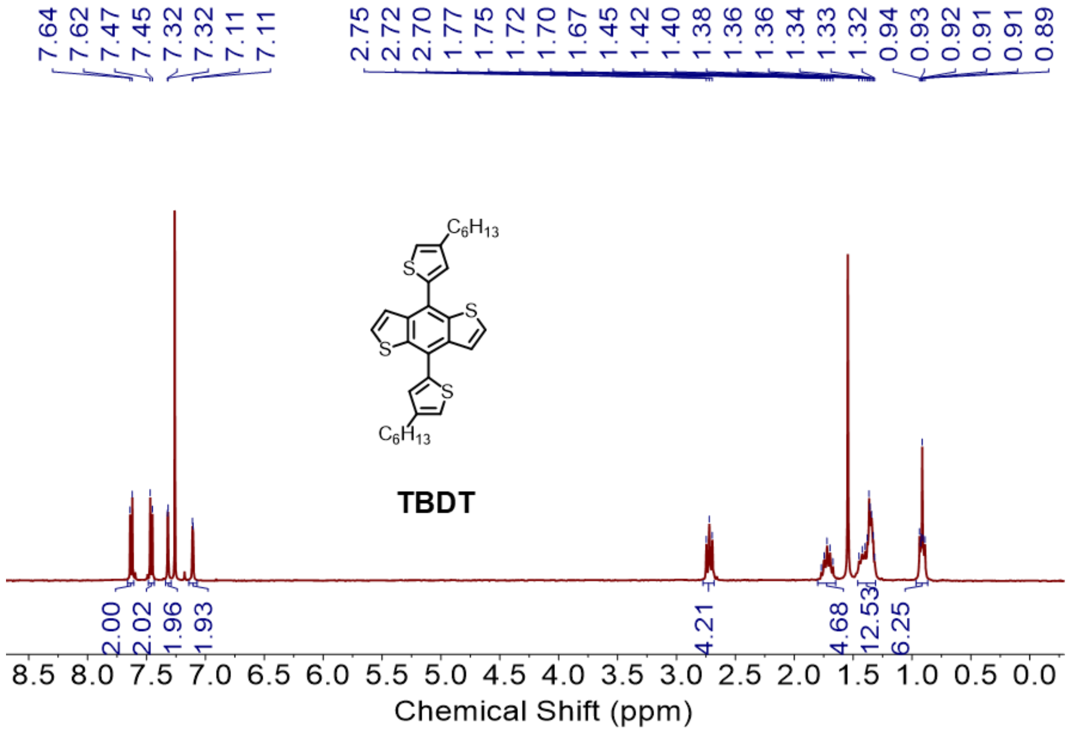

Supplementary Figure 37. ^{1}H NMR ($CDCl_3$, 300 MHz, 25 °C) spectrum of TBDT.

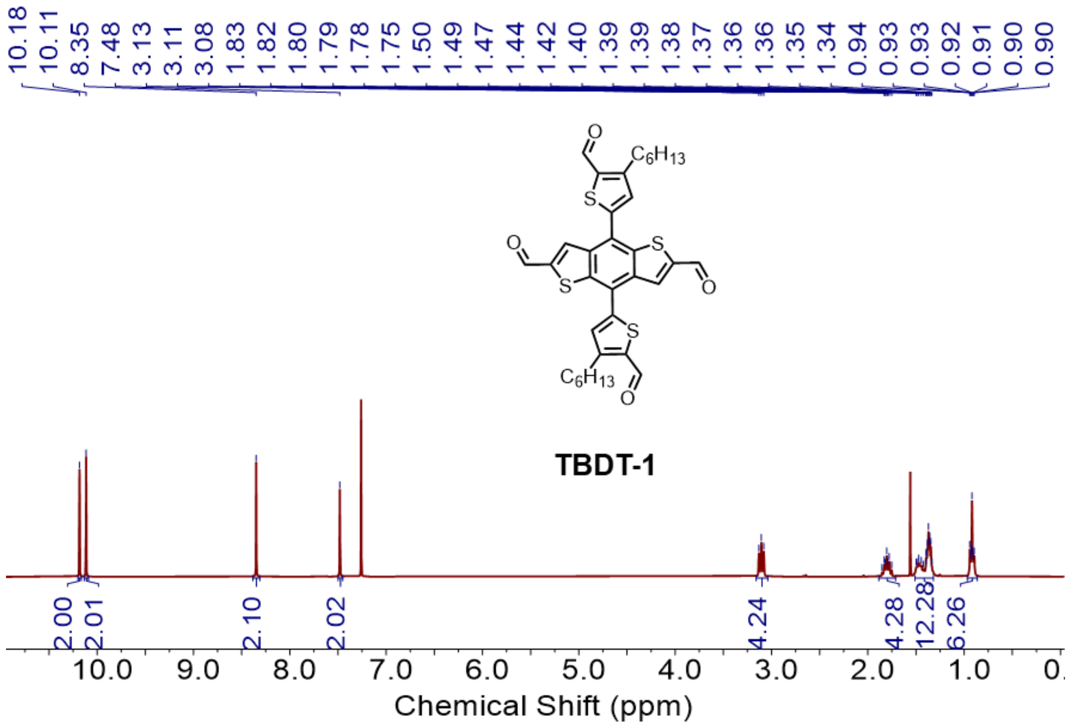

Supplementary Figure 38. ^{1}H NMR ($CDCl_3$, 300 MHz, 25 °C) spectrum of TBDT-1.

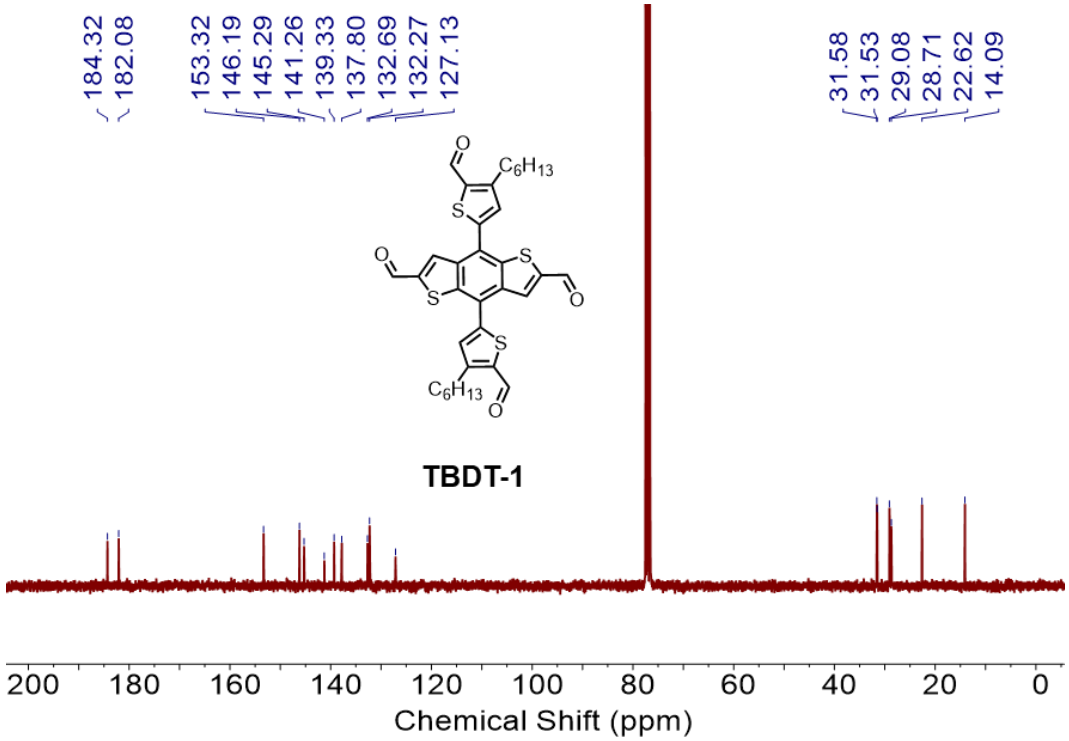

Supplementary Figure 39. ^{13}C NMR ($CDCl_3$, 75 MHz, 25 °C) spectrum of TBDT-1.

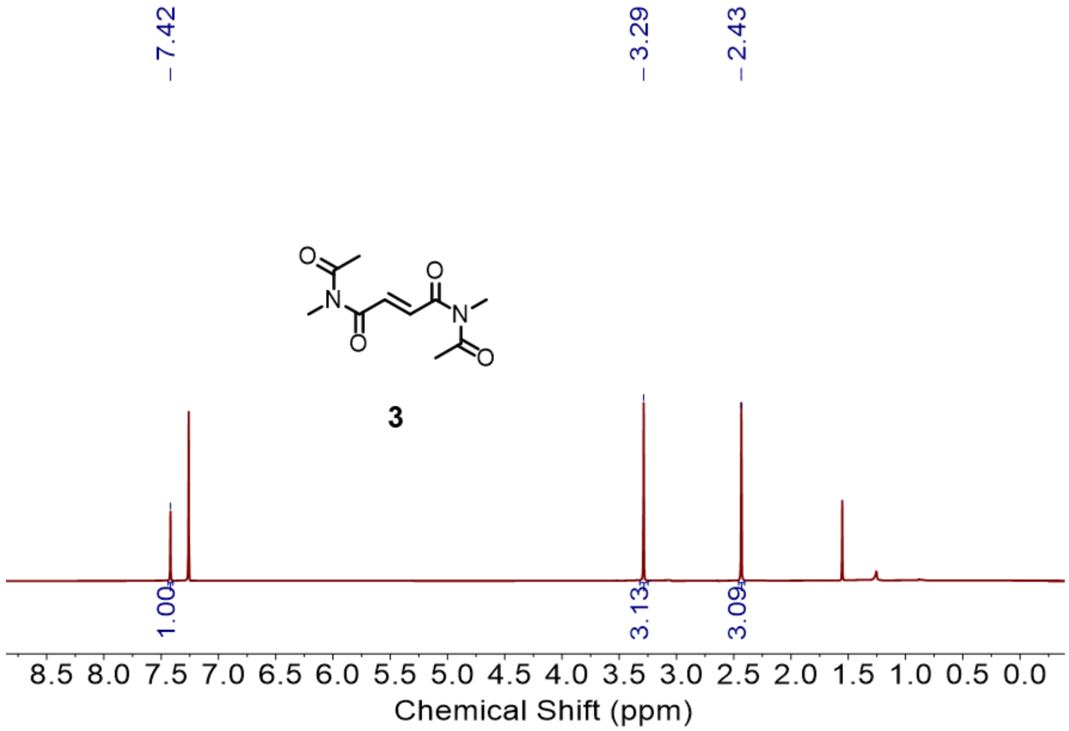

Supplementary Figure 40. ^{1}H NMR ($CDCl_3$, 500 MHz, 25 °C) spectrum of **3**.

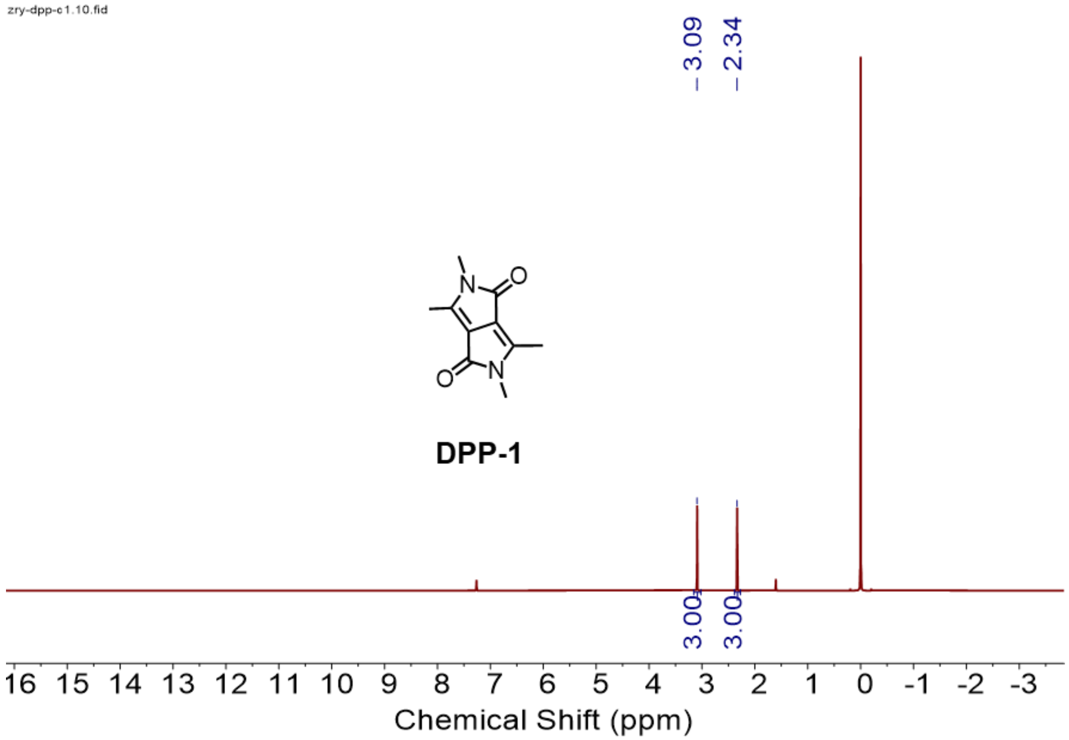

Supplementary Figure 41. ^{1}H NMR ($CDCl_3$, 300 MHz, 25 °C) spectrum of DPP-1.

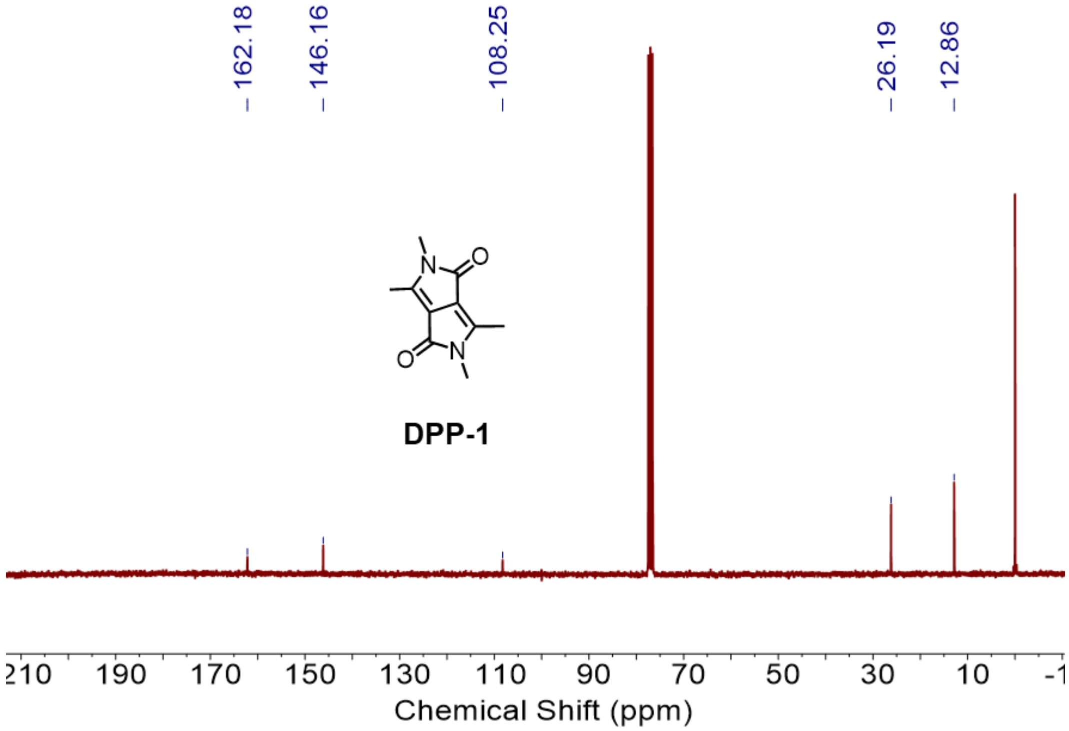

Supplementary Figure 42. ^{13}C NMR ($CDCl_3$, 75 MHz, 25 °C) spectrum of DPP-1.

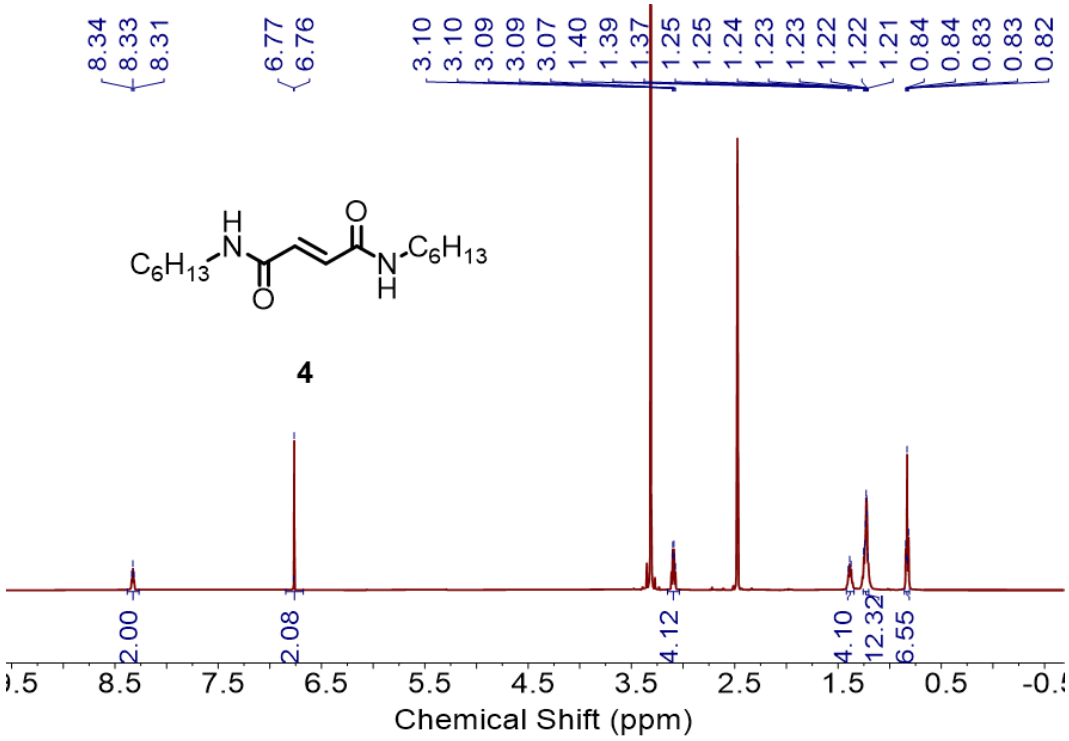

Supplementary Figure 43. ^{1}H NMR (DMSO, 500 MHz, 25 °C) spectrum of **4**.

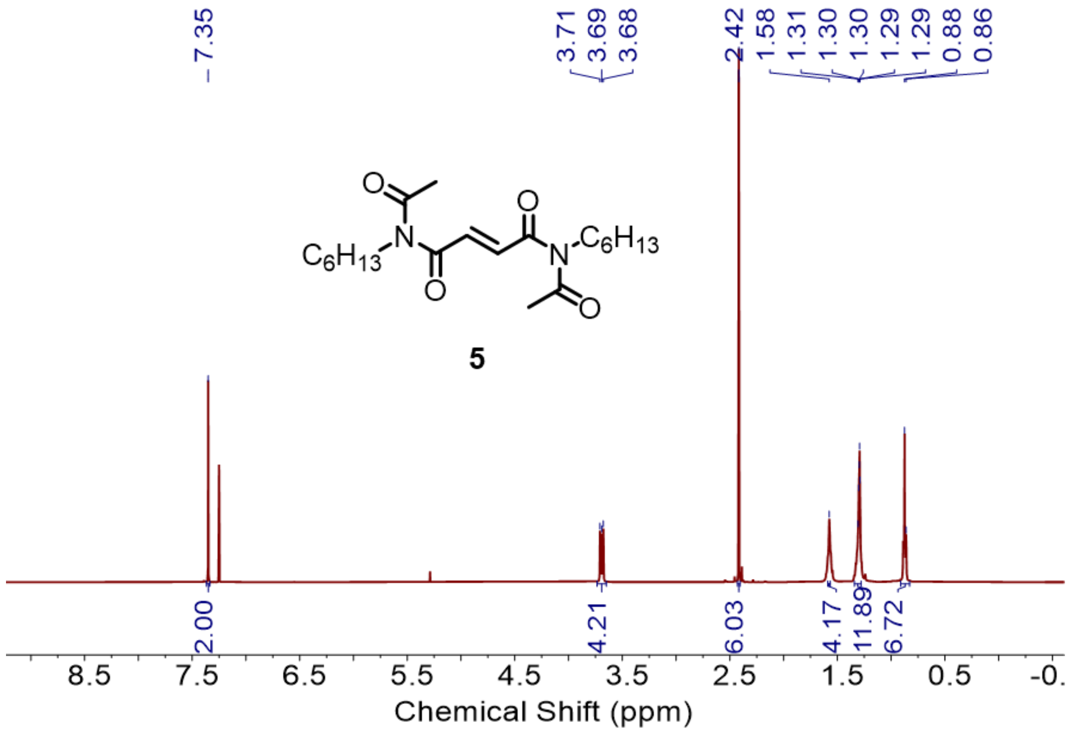

Supplementary Figure 44. ^{1}H NMR ($CDCl_3$, 500 MHz, 25 °C) spectrum of **5**.

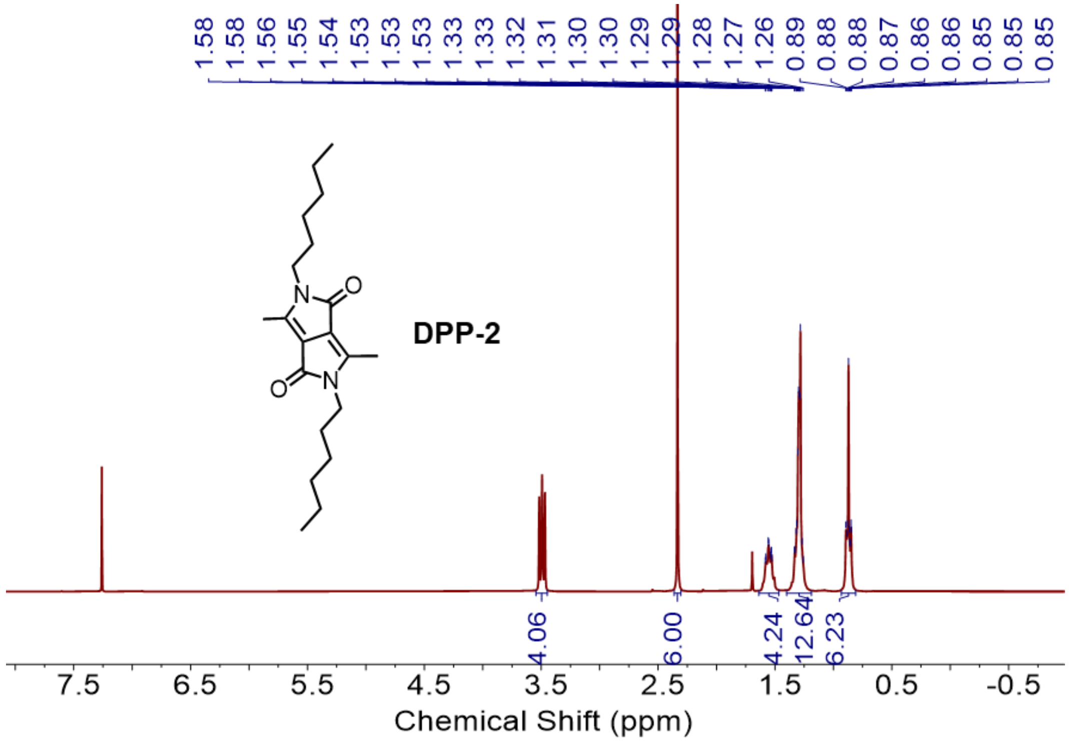

Supplementary Figure 45. ^{1}H NMR ($CDCl_3$, 300 MHz, 25 °C) spectrum of DPP-2.

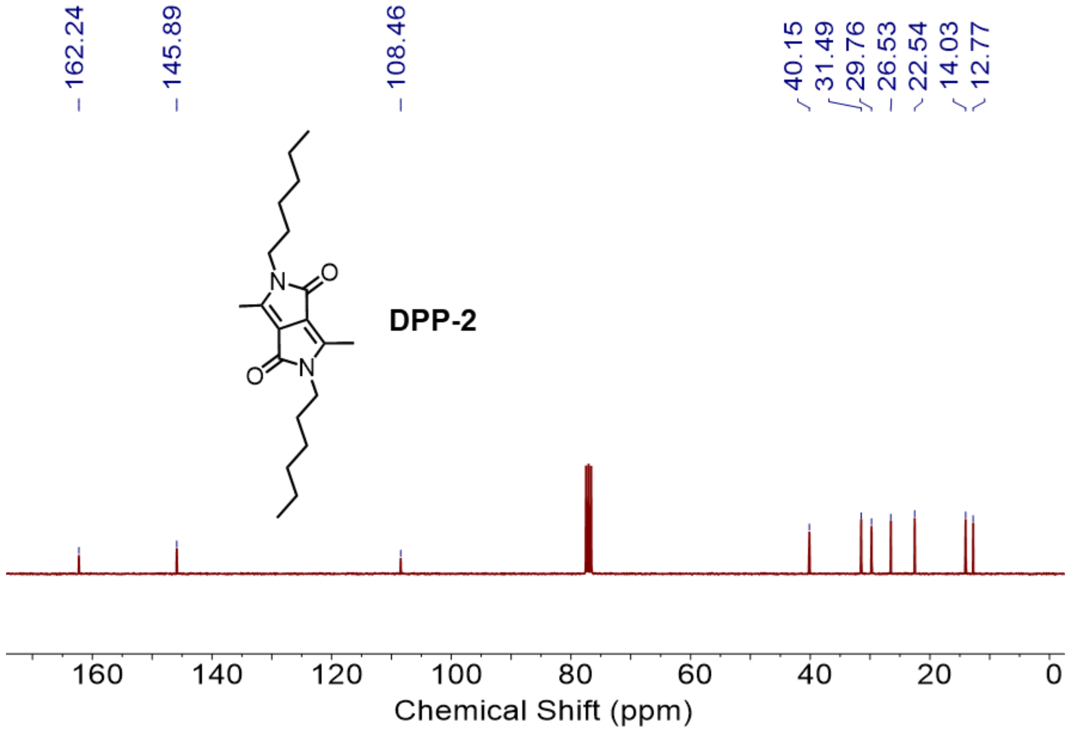

Supplementary Figure 46. ^{13}C NMR ($CDCl_3$, 75 MHz, 25 °C) spectrum of DPP-2.

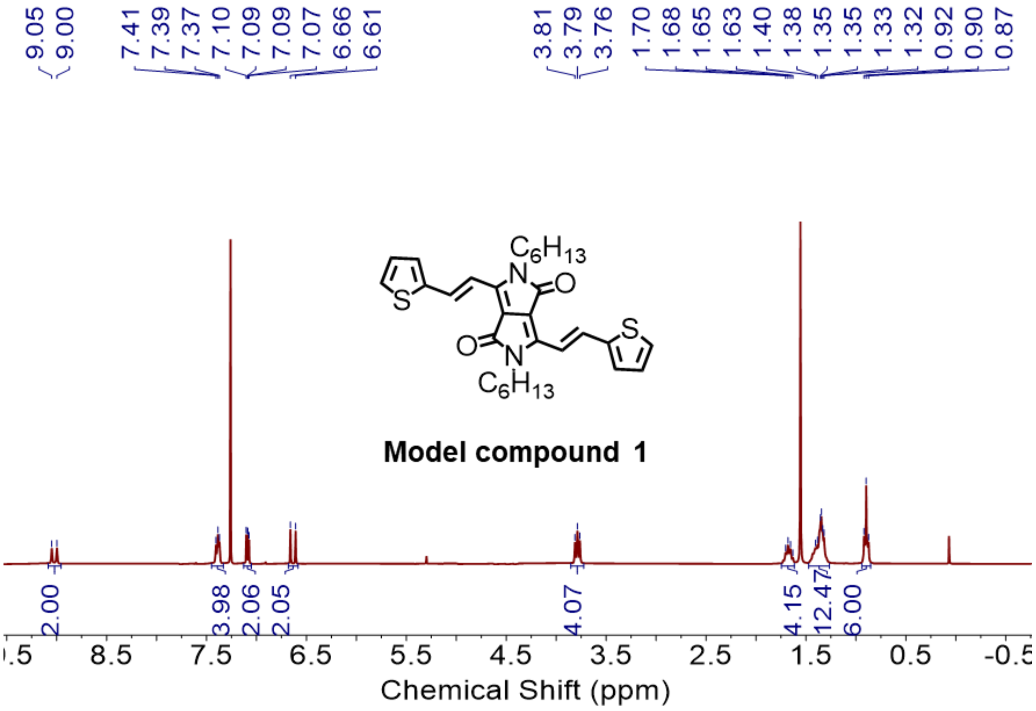

Supplementary Figure 47. ^{1}H NMR ($CDCl_3$, 300 MHz, 25 °C) spectrum of Model compound 1.

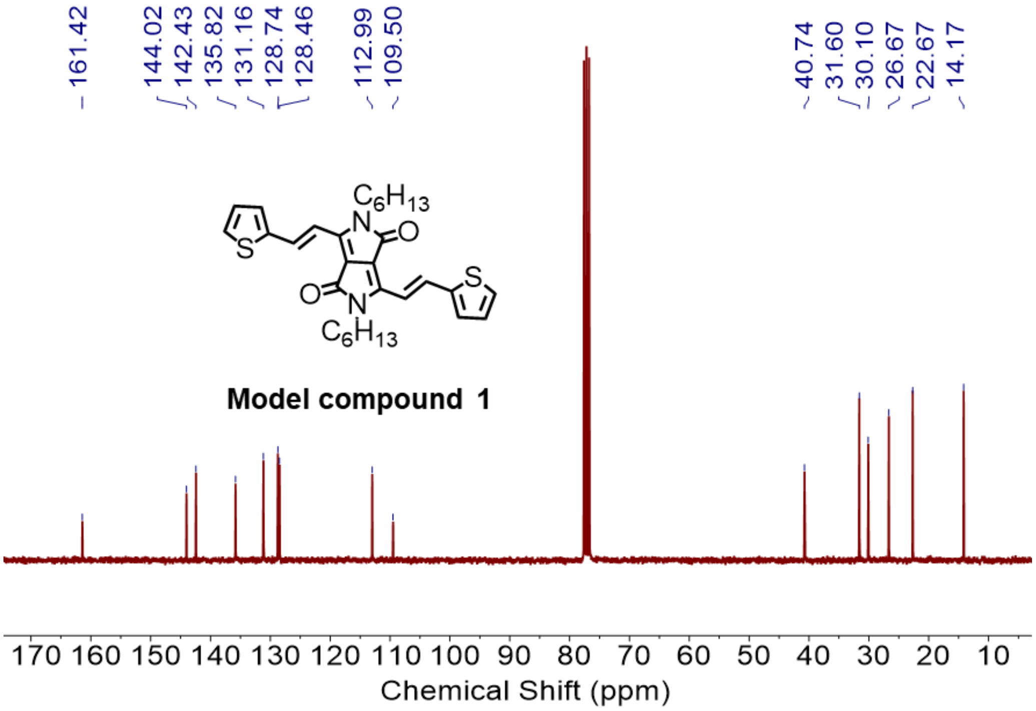

Supplementary Figure 48. ^{13}C NMR ($CDCl_3$, 75 MHz, 25 °C) spectrum of Model compound 1.

Section D. Supplementary Tables

Supplementary Table 1. Starting materials and resources

Chemical Name	Resource
Benzo[1,2-*b*:4,5-*b*']dithiophene-4,8-dione	TCI Deutschland GmbH
n-butyllithium solution (2.5 M in hexanes)	TCI Deutschland GmbH
3-Hexylthiophene	TCI Deutschland GmbH
Fumaryl chloride	Sigma-Aldrich
2.0 M $MeNH_2$ in THF	Sigma-Aldrich
Mesitylene	Acros
1,4-Dioxane	Acros
Isopropenyl acetate	Sigma-Aldrich
Anhydrous tetrahydrofuran	Alfa Aesar
Anhydrous acetone	Alfa Aesar
Acetic acid	Sigma-Aldrich
NH_4OH	Sigma-Aldrich
Pyridinium *p*-toluenesulfonate	TCI Deutschland GmbH
Sodium benzoate	BLD Pharmatech GmbH
Benzoate anhydride	BLD Pharmatech GmbH
1,3-Dimethyl-2-imidazolidinone (DMI)	Sigma-Aldrich
N,N-Dimethylacetamide (DMAc)	Alfa Aesar
1-Methyl-2-pyrrolidinone (NMP)	Sigma-Aldrich

Supplementary Table 2. Condition screening for the synthesis of crystalline 2DPAV-TBDT-DPP-1/2.

No.	**Catalyst**	**Solvent**	**Temperature**	**Crystallinity**
**1**	L-proline/TEA	n-BuOH:*o*-DCB (1:1)	120 °C, 72 h	No polymer
**2**	PTSA・H_2O	n-BuOH:*o*-DCB (1:1)	120 °C, 72 h	No polymer
**3**	Benzoic anhydride/benzoic acid	No solvent	180 °C, 72 h	Moderate
**4**	CH_3COONH_4	Dioxane	120 °C, 72 h	Amorphous
**5**	Benzoic anhydride/benzoic acid	Dioxane:mesitylene (1:1)	150 °C, 72 h	Low
**6**	Benzoic anhydride/benzoic acid	No solvent	200 °C, 72 h	Moderate
**7**	Benzoic anhydride/sodium benzoate	No solvent	200 °C, 72 h	Decent
**8**	Benzoic anhydride	No solvent	200 °C, 72 h	Low
**9**	Sodium benzoate	No solvent	200 °C, 72 h	High
**10**	Acetic anhydride	Acetic anhydride	120 °C, 72 h	Moderate
**11**	L-proline/TEA	No solvent	120 °C, 72 h	No polymer
**12**	Acetic anhydride/sodium benzoate	No solvent	120 °C, 72 h	Decent
**13**	Sodium benzoate	No solvent	120 °C, 72 h	Moderate
**14**	KOH	No solvent	120 °C, 72 h	Moderate
**15**	L-proline/TEA	Toluene/methanol (1:1)	100 °C, 72 h	Amorphous
**16**	DMAP/L-proline	Dioxane:water (9:1)	120 °C, 72 h	Amorphous

Supplementary Table 3. Effective masses of monolayer and layer-stacked 2DPAV-TBDT-DPP-0/1/2.

	Electron			Hole		
Directions	a	b	c	a	b	c
Monolayer 2DPAV-TBDT-DPP-0	**0.072**	0.273	/	**0.159**	0.165	/
Layer-stacked 2DPAV-TBDT-DPP-0	**0.062**	0.111	3.455	0.141	**0.078**	3.457
Monolayer 2DPAV-TBDT-DPP-1	**0.069**	0.140	/	0.155	**0.100**	/
Layer-stacked 2DPAV-TBDT-DPP-1	**0.036**	0.041	1.495	0.132	**0.087**	3.623
Monolayer 2DPAV-TBDT-DPP-2	**0.077**	0.376	/	**0.132**	0.243	/
Layer-stacked 2DPAV-TBDT-DPP-2	**0.069**	0.282	2.93	**0.148**	0.350	7.74

In our studies, we do not distinguish the contribution of electrons and holes. The inferred scattering time is the averaged value with the contribution of both types of charge carriers. Therefore, we take the reduced electron-hole mass by taking the contribution of both electrons and holes into account following:

$$\frac{1}{{m_{e-h}}^{*}} = \frac{1}{m_e^*} + \frac{1}{m_h^*}$$

to infer the final mobility value. In which, the m_e^* *and* m_h^* represent electron and hole effective masses, respectively.

Supplementary Table 4. Summary of charge carrier mobilities of 2DPAV-TBDT-DPP-1/2 and other reported 1D CPs (including graphene nanoribbons) and other organic 2D framework materials (e.g., COFs, MOFs) measured by THz spectroscopy at ambient temperature.

**Sample**	**Theory method**[a]	**Applied *m** (m$_0$)**	**τ (fs)**	***c***	**μ (cm^2 V^{-1} s^{-1})**
**This work**					
2DPAV-TBDT-DPP-1	DFT (*slipped AA*)	0.025	36 ± 5	-0.93 ± 0.02	170 ± 40
2DPAV-TBDT-DPP-2	DFT (*slipped AA*)	0.047	76 ± 4	-0.89 ± 0.02	310 ± 50
**2D conjugated COFs (*conjugated polymers*)**					
2DCP-NiPc[10]	DFT (*AA-s*)	0.137[b]	61 ± 4	−0.72 ± 0.01	219 ± 14 (*r.t.*)
2DCP-CuPc		0.172[b]	69 ± 7	−0.74 ± 0.02	183 ± 19 (*r.t.*)
ZnPc-pz[11]	DFT (*AA-s*)	2.3 (*hole*)	30 ± 4	−0.91 ± 0.02	~2.0
CuPc-pz		2.3 (*hole*)	30 ± 4	−0.97 ± 0.02	~0.7
sp^{2}c-COF[12]	DFTB (*AA*)	0.2[b]	41 ± 5	−0.94 ± 0.02	22.1 ± 2.7
sp^{2}c-COF-6		~0.9[b,c]	~35[c]	N/A	2.3 ± 0.5
sp^{2}c-COF-8		~0.9[b,c]	~10[c]	N/A	<0.1
sp^{2}c-COF-9		~0.9[b,c]	~50[c]	N/A	5.8 ± 0.9
c-HBC-COF[13]	DFTB (*AA*)	0.21[b]	87 ± 5	N/A	44
DBOV-COF[14]	DFTB (*ABC*)	N/A	36 ± 6	−0.85 ± 0.02	0.6 ± 0.1
V-2D-COF-W1[15]	DFT (*AA-s*)	0.63[b]	49 ± 8	N/A	~1.4
V-2D-COF-W3		N/A	37 ± 6	N/A	~10.3
V-2D-COF-W4		N/A	56 ± 8	N/A	~0.6
**Doped 2D conjugated COFs (*conjugated polymers*)**					
ZnPc-pz-I$_2$[16]	DFT (*AA-s*)	2.1 (*hole*)	66 ± 3	−0.98 ± 0.02	6.3
I$_2$-sp^{2}c-COF[12]	N/A	N/A	~85[c]	N/A	51.1 ± 3.1
I$_2$-sp^{2}c-COF-6	N/A	N/A	~70[c]	N/A	5.7 ± 0.3
I$_2$-V-2D-COF-W1[15]	N/A	N/A	110 ± 12	N/A	3.1

I_2-V-2D-COF-W4	N/A	N/A	104 ± 14	N/A	1.2
**2D non-conjugated COFs (*polymers*)**					
CuPc-MIDA-COF[17]	DFTB (*AA*)	~0.6[b,c]	23 ± 13	N/A	13.3
HHTP-MIDA-COF		~1.2[b,c]	16 ± 12	N/A	3.4
**2D conjugated MOFs (*conjugated coordination polymers*)**					
$K_3Fe_2[PcFe\text{-}O_8]$[18]	DFT (*AA-s*)	~1.9[d]	53 ± 8	−0.69 ± 0.02	*15 ± 2*[e]
**1D CPs including graphene nanoribbons**					
FBDOPV-2T[19]			110 ± 7	−0.997	N/A
FBDOPV-2F2T			119 ± 4	−0.996	N/A
FBDOPV-4F2T			71 ± 4	−0.997	N/A
FGNR[20]			28 ± 1	−0.99	*104 ± 3*[e]
cGNR[21]			57 ± 3	−1	*617 ± 32*[e]
cMGNR[22]			36 ± 2	−0.97	2.4
6-CZGNR-(2,1)[23]			29 ± 2	−0.97	~18
6-CGNR-edge[24]			40 ± 3	−0.92	N/A
6-CGNR-cove			35 ± 2	−0.93	N/A
p-AGNR			18 ± 4	−0.88	N/A
9-AGNRs[25]			20 ± 5	−0.72	*352 ± 88*[e]
**Other materials**					
$MoTe_2$ (few-layer)[26]			11 ± 2	0 (Drude)	45 ± 9
Doped BP (nanosheets)[27]			88 ± 3	−0.88	97 ± 3
**2D conjugated and non-conjugated COFs measured by the TRMC rather than THz technique**					
COF-366[28]					8.1
COF-66					3.0
CS-COF[29]					4.2
H_2P-COF[30]					3.5
CuP-COF					0.19
ZnP-COF					0.032/0.016
NiPc COF[31]					1.3

HBC-COF[32]	0.7
2D-NiPc-BTDA COF[33]	0.6
TTF-Ph-COF[34]	0.2
TTF-Py-COF	0.08
2D D-A COF[35]	0.01/0.04

[a]DFT and DFTB represent two typical methods to calculate energy band diagram.

[b]Reduced $m*$ of electron and hole.

[c]Estimated from literature.

[d]Averaged $m*$ of electron and hole.

[e]Calculated by $\mu = \frac{e\tau}{m^*}$ and c was neglected.

Supplementary Table 5. Details of the effective mass calculations for the above-mentioned examples.

Ref.	Calculation method
General	The effective mass tensor of holes (m_h*) and electrons (m_e*) are obtained by the following relation: $$\frac{1}{m^*} = \frac{1}{\hbar^2}\left(\frac{\partial^2 E_n(\vec{\mathbf{k}})}{\partial \mathbf{k_i k_j}}\right) \; (i, j = x,y,z)$$ Where $\hbar$ is the reduced Planck constant; x, y, and z denote the direction in k-space and $E_n(\vec{\mathbf{k}})$ is the dispersion relation of n-th electronic band; $\frac{\partial^2 E_n(\vec{\mathbf{k}})}{\partial \mathbf{k_i k_j}}$ is the second derivative of the CBMs or VBMs with respect to wave vector **k** (can be simply understood as the curvature in the band dispersion)
Ref. 10	**Band structure calculation:** Generalized gradient approximation (GGA) of the exchange-correlation energy in the form of Perdew-Burke-Ernzerhof (PBE) was applied<br>**Fitting method:** Parabolic fitting of the VBM and CBM using SUMO Python toolkit or manually in the case of nearly degenerated electronic states<br>**Effective mass used for mobility calculation:** electron-hole reduced effective mass (m^*) considering averaged effective masses for electrons ($m^*_{(avg)}$) and holes ($m^*_{(avg)}$) $$\frac{1}{m^*} = \frac{1}{m^*_{e(\text{avg})}} + \frac{1}{m^*_{h(\text{avg})}}$$
Ref. 11	**Band structure calculation:** Exchange-correlation functional was treated at GGA level in the form of PBE<br>**Fitting method:** the finite difference method on a five-point stencil<br>**Effective mass used for mobility calculation:** only considered the component m_{zz}* mobility
Ref. 12	**Band structure calculation:** PBE-D3/DZ<br>**Fitting method:** the finite difference method on a five-point stencil<br>**Effective mass used for mobility calculation:** the reduced electron-hole effective mass (m*) $$\frac{1}{m^*} = \frac{1}{m_e} + \frac{1}{m_h}$$
Ref. 13	**Band structure calculation:** DFT calculations were performed using the Vienna ab-initio simulation package (VASP). A GGA in the form of PBE is used for the exchange and correlation functional<br>**Fitting method:** Parabolic fitting

	**Effective mass used for mobility calculation:** To take into account the effective mass contribution from electrons and holes for mobility, they further use the reduced effective mass of charge carriers m* following: $$\frac{1}{m^*} = \frac{1}{m_e^*} + \frac{1}{m_h^*}$$
Ref. 14	**Band structure calculation:** Geometries were calculated using Density Functional Tight Binding (DFTB) as implemented in DFTB+ version 20.1. Following geometry optimization, the band structure, Density of States (DOS) and effective mass were calculated using 3rd order DFTB and the 3ob-3-1 parameter set<br>**Fitting method:** Not mentioned<br>**Effective mass used for mobility calculation:** 3rd order DFTB and the 3ob-3-1 parameter set
Ref. 15	**Band structure calculation:** Calculation was carried out under *k*-point grid of 3×3×6, in implement with the TIER1 basis set and tight integration grid. Herd−Scuseria−Ernzerhof hybrid functional (HSE06) was used with 25% Hartree–Fock exchange and a screening parameter of 0.11 $bohr^{-1}$. Many-body method was adopted to correct the long-range van der Waals dispersion<br>**Fitting method:** Not mentioned<br>**Effective mass used for mobility calculation:** the reduced electron-hole effective mass calculated by: $$\frac{1}{m^*} = \frac{1}{m_e} + \frac{1}{m_h}$$
Ref. 16	The same as Ref. 10
Ref. 17	The same as Ref. 11
Ref. 18	**Band structure calculation:** Generalized gradient approximation (GGA) of the exchange-correlation energy in the form of Perdew-Burke-Ernzerhoff (PBE) was applied<br>**Fitting method:** Not mentioned<br>**Effective mass used for mobility calculation:** An electron-hole averaged effective mass
Ref. 19	**Band structure calculation:** geometries of monomer were optimized at the B3LYP/6-311G(d,p) level<br>**Fitting method:** Not mentioned<br>**Effective mass used for mobility calculation:** not applied
Ref. 20	**Band structure calculation:** DFT level of theory with the HSE06 functional and 6-31G(d) basis set

	**Fitting method:** Not mentioned<br>**Effective mass used for mobility calculation:** From the band dispersion, they computed the reduced mass around the VBM and CBM, as well as the VBM and CBM orbitals. Furthermore, they estimated the effective reduced mass of charge carriers m*, by considering the averaged values for both charges, with the equation:<br>$$\frac{1}{m^*} = \frac{1}{m_e^*} + \frac{1}{m_h^*}$$
Ref. 21	**Band structure calculation:** All computations have been performed with the Gaussian16 software, considering the DFT level of theory and the range-separated screened HSE hybrid functional with the Pople's 631G(d) basis set<br>**Fitting method:** Not mentioned<br>**Effective mass used for mobility calculation:** As both the electron and hole contribute to the photoconductivity, they approximate the mass by the averaged value of both charges and use the reduced mass of charge carriers calculated by:<br>$$\frac{1}{m^*} = \frac{1}{m_e^*} + \frac{1}{m_h^*}$$<br>The effective masses of the electron and the hole have been calculated by DFT calculation performed on the optimized cGNR structure
Ref. 22	The same as Ref. 21
Ref.23	**Band structure calculation:** DFT level of theory with the HSE functional and 6-31G(d) basis set, using the Gaussian16 suite of programs. For the infinite polymer, periodic boundary conditions (PBC) were applied<br>**Fitting method:** Not mentioned<br>**Effective mass used for mobility calculation:** Not mentioned
Ref.24	**Band structure calculation:** The electronic structure was calculated at the DFT level using the screened exchange hybrid exchange−correlation functional HSE06 and the standard 6-31G* basis set<br>**Fitting method:** Not mentioned<br>**Effective mass used for mobility calculation:** Not mentioned
Ref.25	**Band structure calculation:** Not mentioned<br>**Fitting method:** Not mentioned<br>**Effective mass used for mobility calculation:** Use the reported effective mass
Ref.26	Not mentioned
Ref.27	**Band structure calculation:** The Perdew-Burke-Ernzhof (PBE) exchange-correlation functional and the projector-augmented wave (PAW) method were employed for structural optimization<br>**Fitting method:** Not mentioned

	**Effective mass used for mobility calculation:** use effective mass of charge carriers in the 5-layer BP obtained from the literature
Ref. 28–35	TRMC, rather than THz technique, doesn't need effective mass values
This work	**Band structure calculation:** The electronic properties of the 2D PAVs were calculated using density functional theory (DFT) with the Vienna Ab Initio Simulation Package (VASP 5.4.4), implementing the projector-augmented wave (PAW) method and the Perdew-Burke-Ernzerhof (PBE) functional<br>**Fitting method:** Parabolic fitting<br>**Effective mass used for mobility calculation:** In our studies, we do not distinguish the contribution of electrons and holes. The inferred scattering time is the averaged value with the contribution of both types of charge carriers. Therefore, we take the reduced electron-hole mass by taking the contribution of both electrons and holes into account following:<br>$$\frac{1}{m_{e-h}{}^{*}} = \frac{1}{m_e^*} + \frac{1}{m_h^*}$$<br>to infer the final mobility value. In which, the $m_e^*\ and\ m_h^*$ represent electron and hole effective masses, respectively

Supplementary Table 6. Comparison of $FWHM_{100}$ of the representative 2D COFs with different linkages.

Sample	Linkage	$FWHM_{100}$ (degree)	Reference
g-$C_{40}N_3$-COF	C=C	0.305	Nat. Commun. 2019, 10, 2467
g-$C_{31}N_3$-COF	C=C	0.884	Nat. Commun. 2019, 10, 2467
g-$C_{37}N_3$-COF	C=C	0.934	Nat. Commun. 2019, 10, 2467
v-2D-COF-NS1	C=C	0.7	ACS Catal. 2023, 13, 1089−1096
COF-1	Triazine	0.3	Angew. Chem. Int. Ed. 2019, 58, 13753
g-DZPH-COF	C=C	0.19	Angew. Chem. Int. Ed. 2024, 63, e202402446
g-DZTA-COF	C=C	0.26	Angew. Chem. Int. Ed. 2024, 63, e202402446
V-2D-COFs	C=C	0.96-1.03	Angew. Chem. Int. Ed. 2022, 61, e202209762
ivCOF-1-Br	C=C	0.783	Angew. Chem. Int. Ed. 2021, 60, 13614−13620
ivCOF-2-Br	C=C	0.528	Angew. Chem. Int. Ed. 2021, 60, 13614−13620
ivCOF-2-I	C=C	0.635	Angew. Chem. Int. Ed. 2021, 60, 13614
g-COF-DPP-1	C=C	0.428	Angew. Chem. Int. Ed. 2025, 64, e202417805
g-COF-DPP-2	C=C	0.535	Angew. Chem. Int. Ed. 2025, 64, e202417805

NKCOF-28	imide	0.792	Chem, 2023, 9, 2178−2193
LZU-600	pyrano[4,3-*b*]pyridine	0.46	J. Am. Chem. Soc. 2022, 144, 6594−6603
LZU-601	pyrano[4,3-*b*]pyridine	0.28	J. Am. Chem. Soc. 2022, 144, 6594−6603
LZU-602	pyrano[4,3-*b*]pyridine	0.50	J. Am. Chem. Soc. 2022, 144, 6594−6603
LZU-603	pyrano[4,3-*b*]pyridine	0.40	J. Am. Chem. Soc. 2022, 144, 6594−6603
TFPPy-PDA-COF	imine	0.38	J. Am. Chem. Soc. 2022, 144, 9624−9633
TPB-DMTP-COF	imine	0.39	Nat. Chem. 2015, 7, 905−912
[*S*-Py]$_{0.17}$-TPB-DMTP-COF	imine	0.437	Nat. Chem. 2015, 7, 905−912
sc-COF$_{TP\text{-}Py}$	imine	0.34	Nat. Commun. 2021, 12, 5077
NKCOF-10	C=C	0.81	Nat. Commun. 2021, 12, 1982
QL-COF-2	4-carboxyl-quinoline	1.04	Nat. Commun. 2022,13, 2615
2DPAV-TBDT-DPP-1	C=C	0.39	*This work*

Supplementary Table 7. Comparison of band gaps or absorption maxima of the reported 2D CPs.

Sample	Absorption Maxima (nm)[a]	Optical Band Gap (eV)[b]	Reference
2D CPs without DPP units			
TPB-TFB COF	~410	2.6	*J. Am. Chem. Soc*. 2022, 144, 7489−7496
c-HBC-COF	~500	2.18	*J. Am. Chem. Soc. 2022,* 144, 5042–5050
COF-Nap	~450	2.28	*J. Am. Chem. Soc*. 2023, 145, 26871–26882
sp^2c-COF	~500	2.05	*Nat. Commun*. 2018, 9, 4143
V-2D-COF-W1	~450	2.18	*Angew. Chem. Int. Ed*. 2022, 61, e202209762
V-2D-COF-W3	~450	2.26	*Angew. Chem. Int. Ed*. 2022, 61, e202209762
V-2D-COF-W4	~450	2.21	*Angew. Chem. Int. Ed*. 2022, 61, e202209762
2DPAV-BDT-BP	~500	1.9	*Angew. Chem. Int. Ed*. 2023, 62, e202305978
2DPAV-BDT-BT	~550	1.62	*Angew. Chem. Int. Ed*. 2023, 62, e202305978
2DPAV-TBDT-IT	~890	1.15	*Angew. Chem. Int. Ed. 2025, e202504302*
2DCP-CuPc	~700	1.28	*Nat. Mater*. 2023, 22, 880−887
2DCP-NiPc	~700	1.33	*Nat. Mater*. 2023, 22, 880−887
COF-Az	~800	1.37	*J. Am. Chem. Soc*. 2023, 145, 26871–26882
2D CPs containing DPP units			
g-COF-DPP-1	~710	1.08	*Angew. Chem. Int. Ed*. 2025, 64, e202417805

g-COF-DPP-2	~690	1.02	*Angew. Chem. Int. Ed.* 2025, 64, e202417805
DPP-TAPP-COF	~670	-	*Angew. Chem. Int. Ed.* 2018, 57,846–850
TpDPP-Py COFs	~750	1.38	*Nat. Commun.* 2024, 15, 4856
DPP-Py COFs	~600	-	*Nat. Commun.* 2024, 15, 4856
DPP-TPP-COF	~673	1.63	*Small 2024, 2402993*
DPP-TBB-COF	~666	1.64	*Small 2024, 2402993*
2DPAV-TBDT-DPP-1	**~910**	**1.0**	***This work***
2DPAV-TBDT-DPP-2	**~837**	**1.2**	***This work***

[a]Estimated from literature. [b]Extracted from the literature.

Supplementary Table 8. Summary of the calculated values of effective masses and mobilities for electron and hole in 2DPAV-TBDT-DPP-0.

Direction	Hole			Electron		
	Effective mass (m_0)	Relaxation time (s)	Mobility[a] ($cm^2 V^{-1} s^{-1}$)	Effective mass (m_0)	Relaxation time (s)	Mobility[a] ($cm^2 V^{-1} s^{-1}$)
Intralayer	0.078	1.52×10^{-14}	110.79	0.062	1.97×10^{-13}	1801.62
Interlayer	3.457		6.99	3.455		37.67

[a]The mobility was calculated using Boltzmann transport equation (BTE) in the relaxation time approximation.[36, 37, 38]

Supplementary Table 9. Summary of the *n*-doping behavior of DPP-based linear conjugated polymers and 2D COFs.

Linear conjugated polymers			
Sample	*n*-dopants	Conductivity (S/cm)	Reference
P(TDPP-CT2)	*N*-DMBI	0.39	*J. Am. Chem. Soc.* 2019, 141, 20215−20221
P(PzDPP-CT2)	*N*-DMBI	8.4	*J. Am. Chem. Soc.* 2019, 141, 20215−20221
P(PzDPP-2FT)	$CoCp_2$	129	*Nat. Commun.* 2021, 12, 5723
P(PzDPP-2FT)	*N*-DMBI	43.3	*Nat. Commun.* 2021, 12, 5723
P(BTP-DPP)	$(RuCp^*mes)_2$	0.45	*Chem. Mater.* 2017, 29, 22, 9742–9750
pDFSe	*N*-DMBI	62.6	*Angew. Chem. Int. Ed.* 2024, 63, e202409018
ThDPP-CNBTz	*N*-DMBI	50.6	*Angew. Chem. Int. Ed.* 2024, 63, e202402642
PTz-5-DPP	*N*-DMBI	8	*Angew. Chem. Int. Ed.* 2023, 62, e202219262
2D COFs			
DPP-TPP-COF	TDAE	5.1×10^{-5}	*Small* 2024, 2402993
DPP-TBB-COF	TDAE	1.2×10^{-5}	*Small* 2024, 2402993

Section E. Supporting References

1. Liu, Y. et al. A thiophene backbone enables two-dimensional poly(arylene vinylene)s with high charge carrier mobility. *Angew. Chem. Int. Ed.* **62**, e202305978 (2023).

2. Kresse, G. & Furthmüller, J. Efficient iterative schemes for ab initio total-energy calculations using a plane-wave basis set. *Phys. Rev. B* **54**, 11169-11186 (1996).

3. Blöchl, PE. Projector augmented-wave method. *Phys. Rev. B* **50**, 17953-17979 (1994).

4. Perdew, JP, Burke, K. & Ernzerhof, M. Generalized gradient approximation made simple. *Phys. Rev. Lett.* **77**, 3865-3868 (1996).

5. Grimme, S. et al. A consistent and accurate ab initio parametrization of density functional dispersion correction (DFT-D) for the 94 elements H-Pu. *J. Chem. Phys.* **132**, 154104 (2010).

6. Momma, K. & Izumi, F. VESTA 3 for three-dimensional visualization of crystal, volumetric and morphology data. *J. Appl. Crystallogr.* **44**, 1272-1276 (2011).

7. Lee, SM. et al. Horizontal-, vertical-, and cross-conjugated small molecules: conjugated pathway-performance correlations along operation mechanisms in ternary non-fullerene organic solar cells. *Small* **16**, e1905309 (2020).

8. Dou, J-H. et al. Atomically precise single-crystal structures of electrically conducting 2D metal–organic frameworks. *Nat. Mater.* **20**, 222-228 (2020).

9. Massiot, D. et al. Modelling one- and two-dimensional solid-state NMR spectra. *Magn. Reson. Chem.* **40**, 70-76 (2002).

10. Wang, M. et al. Exceptionally high charge mobility in phthalocyanine-based poly(benzimidazobenzophenanthroline)-ladder-type two-dimensional conjugated polymers. *Nat. Mater.* **22**, 880-887 (2023).

11. Wang, M. et al. Unveiling Electronic Properties in Metal–Phthalocyanine-Based Pyrazine-Linked Conjugated Two-Dimensional Covalent Organic Frameworks. *J. Am. Chem. Soc.* **141**, 16810-16816 (2019).

12. Jin, E. et al. Module-Patterned Polymerization towards Crystalline 2D sp2-Carbon Covalent Organic Framework Semiconductors. *Angew. Chem. Int. Ed.* **61**, e202115020 (2022).

13. Xing, G. et al. Nonplanar Rhombus and Kagome 2D Covalent Organic Frameworks from Distorted Aromatics for Electrical Conduction. *J. Am. Chem. Soc.* **144**, 5042-5050 (2022).

14. Jin, E. et al. A Nanographene-Based Two-Dimensional Covalent Organic Framework as a Stable and Efficient Photocatalyst. *Angew. Chem. Int. Ed.* **61**, e202114059 (2022).

15. Liu, Y. et al. Vinylene-Linked 2D Conjugated Covalent Organic Frameworks by Wittig Reaction. *Angew. Chem. Int. Ed.* **134**, e202209762 (2022).

16. Wang, M. et al. High-Mobility Semiconducting Two-Dimensional Conjugated Covalent Organic Frameworks with p-Type Doping. *J. Am. Chem. Soc.* **142**, 21622-21627 (2020).

17. Jin, E. et al. Exceptional electron conduction in two-dimensional covalent organic frameworks. *Chem* **7**, 3309-3324 (2021).

18. Yang, C. et al. A semiconducting layered metal-organic framework magnet. *Nat. Commun.* **10**, 3260 (2019).

19. Wang, Z-Y. et al. Correlating Charge Transport Properties of Conjugated Polymers in Solution Aggregates and Thin-Film Aggregates. *Angew. Chem. Int. Ed.* **60**, 20483-20488 (2021).

20. Yao, X. et al. Synthesis of Nonplanar Graphene Nanoribbon with Fjord Edges. *J. Am. Chem. Soc.* **143**, 5654-5658 (2021).

21. Niu, W. et al. A Curved Graphene Nanoribbon with Multi-Edge Structure and High Intrinsic Charge Carrier Mobility. *J. Am. Chem. Soc.* **142**, 18293-18298 (2020).

22. Yang, L. et al. Solution Synthesis and Characterization of a Long and Curved Graphene Nanoribbon with Hybrid Cove–Armchair–Gulf Edge Structures. *Adv. Sci.* **9**, 2200708 (2022).

23. Wang, X. et al. Cove-Edged Graphene Nanoribbons with Incorporation of Periodic Zigzag-Edge Segments. *J. Am. Chem. Soc.* **144**, 228-235 (2022).

24. Ivanov, I. et al. Role of Edge Engineering in Photoconductivity of Graphene Nanoribbons. *J. Am. Chem. Soc.* **139**, 7982-7988 (2017).

25. Chen, Z. et al. Chemical Vapor Deposition Synthesis and Terahertz Photoconductivity of Low-Band-Gap N = 9 Armchair Graphene Nanoribbons. *J. Am. Chem. Soc.* **139**, 3635-3638 (2017).

26. Zheng, W, Bonn, M. & Wang, HI. Photoconductivity Multiplication in Semiconducting Few-Layer MoTe2. *Nano Lett.* **20**, 5807-5813 (2020).

27. Shi, H. et al. Molecularly Engineered Black Phosphorus Heterostructures with Improved Ambient Stability and Enhanced Charge Carrier Mobility. *Adv. Mater.* **33**, 2105694 (2021).

28. Wan, S. et al. Covalent Organic Frameworks with High Charge Carrier Mobility. *Chem. Mater.* **23**, 4094-4097 (2011).

29. Guo, J. et al. Conjugated organic framework with three-dimensionally ordered stable structure and delocalized pi clouds. *Nat Commun* **4**, 2736 (2013).

30. Feng, X. et al. High-rate charge-carrier transport in porphyrin covalent organic frameworks: switching from hole to electron to ambipolar conduction. *Angew Chem Int Ed Engl* **51**, 2618-2622 (2012).

31. Ding, X. et al. Synthesis of metallophthalocyanine covalent organic frameworks that exhibit high carrier mobility and photoconductivity. *Angew Chem Int Ed Engl* **50**, 1289-1293 (2011).

32. Dalapati, S. et al. Rational design of crystalline supermicroporous covalent organic frameworks with triangular topologies. *Nat Commun* **6**, 7786 (2015).

33. Ding, X. et al. An n-channel two-dimensional covalent organic framework. *J Am Chem Soc* **133**, 14510-14513 (2011).

34. Jin, S. et al. Two-dimensional tetrathiafulvalene covalent organic frameworks: towards latticed conductive organic salts. *Chemistry* **20**, 14608-14613 (2014).

35. Feng, X. et al. An Ambipolar Conducting Covalent Organic Framework with Self-Sorted and Periodic Electron Donor-Acceptor Ordering. *Advanced Materials* **24**, 3026-3031 (2012).

36. Bardeen, J. & Shockley, W. Deformation Potentials and Mobilities in Non-Polar Crystals. *Phys. Rev.* **80**, 72-80 (1950).

37. Xi, J. et al. Tunable Electronic Properties of Two-Dimensional Transition Metal Dichalcogenide Alloys: A First-Principles Prediction. *J. Phys. Chem. Lett.* **5**, 285-291 (2014).

38. Long, M-Q. et al. Theoretical Predictions of Size-Dependent Carrier Mobility and Polarity in Graphene. *J. Am. Chem. Soc.* **131**, 17728-17729 (2009).
